# Design, synthesis, and catalytic performance of modified graphene oxide based on a cobalt complex as a heterogenous catalyst for the preparation of aminonaphthoquinone derivatives†

**DOI:** 10.1039/d1ra01790j

**Published:** 2021-05-11

**Authors:** Mahnaz Mirheidari, Javad Safaei-Ghomi

**Affiliations:** Department of Organic Chemistry, Faculty of Chemistry, University of Kashan P.O. Box 87317-51167 Kashan I. R. Iran safaei@kashanu.ac.ir

## Abstract

We are reporting a functionalized graphene oxide catalyst developed by modifying graphene oxide surface using the covalent attachment of an amino-functionalized SiO_2_ sphere/cobalt complex. Silica network has special characteristics including mechanical strength, high thermal and chemical stability with good dispersion in solvents. The silica/graphene oxide mixture provides improved properties and extends the scope of application. Graphene oxide was functionalized by spherical silica with the help of hybrid silane-containing nitrogen to coordinate with Co(ii) for increasing the catalytic activity. The catalyst was characterized by Fourier Transform Infrared (FT-IR) spectroscopy, powder X-ray diffraction (XRD), Energy Dispersive X-ray (EDX), Scanning Electron Microscopy (SEM), Raman spectroscopy, and Thermal Gravimetric (TGA) analyses. The catalyst showed high catalytic activity for multi-component reactions in the synthesis of aminonaphthoquinones in ethanol solvent. The catalyst's ability to improve the yield (96–98%), reduce the reaction time (5–8 min), and recycling ability are important benefits for the catalyst.

## Abstract 2

GO@f-SiO_2_@Co is a heterogenous catalyst composed of spherical silica particles grafted on the surface of graphene oxide with ethylenediamine ligands and coordination with Co(ii). We assessed the activity of the catalyst for the synthesis of aminonaphthoquinones.

## Graphical abstract

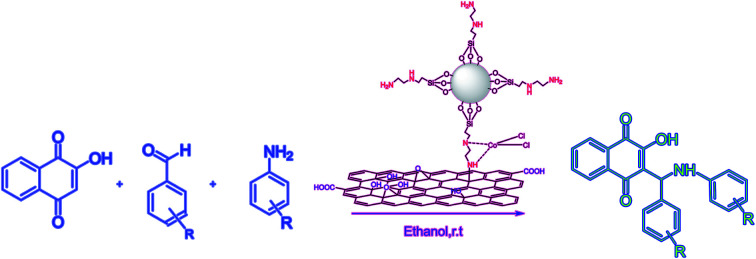

## 1. Introduction

Graphene is one form of carbon material with 2D layered structure that is considered as an excellent catalyst support in recent decades. Geim and Novoselov explored a single layer of graphene through crystal graphite in 2004 for the first time.^1^ Graphene has different applications in various fields including solar cells,^2^ polymer nanocomposites,^3^ and biological nanocomposites^4^ due to its elasticity, chemical stability, and high specific surface area as well as electrical and heat conductivity.^5–7^

Graphene oxide (GO) is the oxidised form of graphene, which has a two-dimensional monolayer honeycomb structure. It is a good precursor material for fabrication in large quantities due to its low-cost and easily scalable synthesis process. In addition, modification of graphene oxide is simple due to chemical interaction between oxygen functional groups on the surface, making it a desirable support in chemical reactions.^8–10^ Several methods have been developed for GO synthesis, however, Hummers' method is the most well-known approach.^11^

The oxygen-containing functional groups that are distributed over the GO surface can act as an active site, and can be attacked by nucleophiles to change the surface of graphene sheets. Therefore, graphene oxide is widely used as a catalyst support.^12,13^ Functionalized graphene oxide has several applications in biomedical, electrochemical, and chemical fields.^14^ In addition, functionalized GO with various metals has been extensively used for organic transformations.^15,16^

As far as we know, among different surface modifying agents, polysiloxane-like silane coupling agents are often applied to obtain modified graphene sheets.^17,18^ During the coupling procedure, the covalent bonds between the functional groups of silane with graphene oxide lead to increased graphene oxide performance in the catalytic field and deliver multifunctional characteristics.^19–21^ Silica (SiO_2_) increases the corrosion resistance and thermal resistance of graphene oxide, which may improve the loading of catalytically active sites, having significant application potential in different fields.^22–27^ In addition, cobalt has obtained more attention because of being an earth-abundant metal, catalytic activities,^28,29^ and photocatalytic H_2_ production.^30^

2-Hydroxy-1,4-napthoquinone (lawsone) has been known for almost 4000 years, which is present in the leaves of the henna plant, and it is a significant source of natural dyes.^31^ The compounds containing the naphthoquinone structure received a great deal of attention because of various biological features such as molluscicidal,^32^ antitumor,^33^ antifungal, and antibacterial^34^ activities as well as fluorescence behavior.^35^

Research shows that the incorporation of nitrogen atom containing functional groups such as amino group or nitrogen atom into the naphthoquinone framework often improves anticancer,^36^ molluscicidal,^37^ and antibacterial activities.^38^

**Scheme 1:**
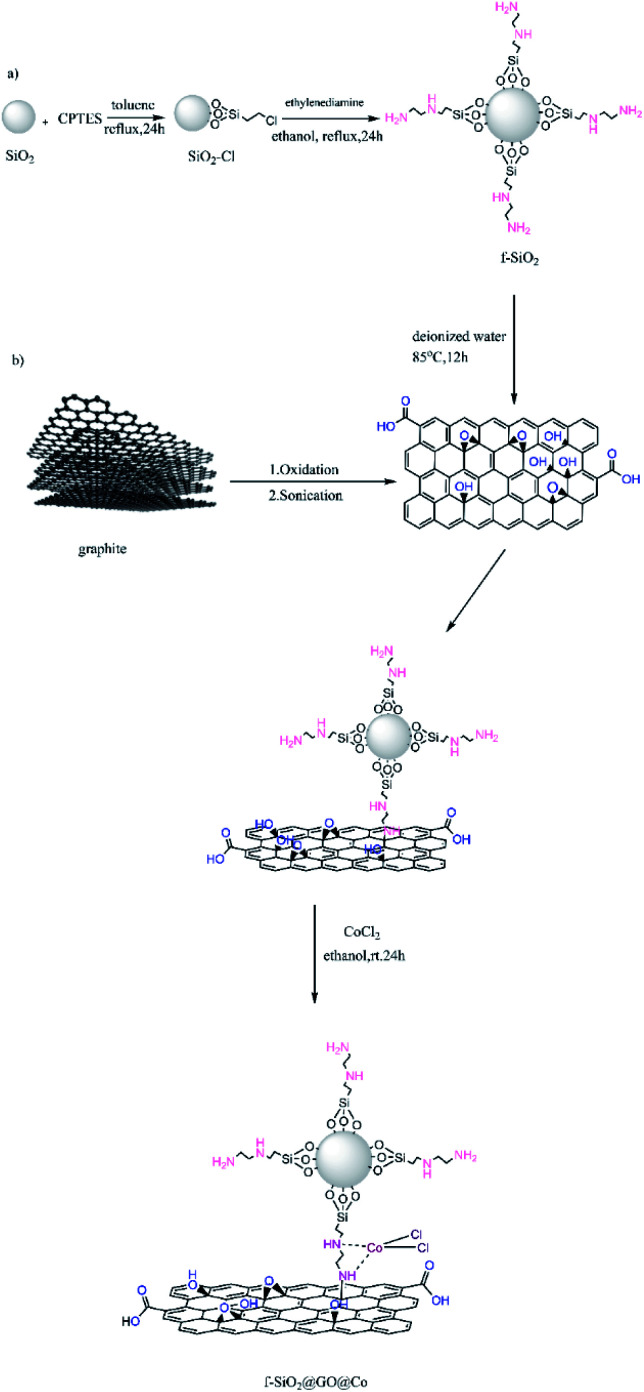
Different steps of synthesis of f-SiO_2_@GO@Co.

Considering the above-mentioned properties of graphene oxide, in the current study, the GO surface was activated with amino-functionalized SiO_2_ containing binding sites for coordination to Co(ii) for increasing the catalytic activity. The produced catalyst as the highly efficient heterogeneous catalyst was used for the synthesis of aminonaphthoquinone derivatives through a three-component condensation reaction of lawsone, aromatic aldehydes, and aromatic amines in the presence of ethanol at room temperature (Scheme 1).

## 2. Experimental

### 2.1. General

All materials were commercially purchased from Merck and Sigma-Aldrich. Melting points of the products were recorded by an Electrothermal 9200 system. Fourier transform infrared measurements were carried out on a Magna 550 instrument by using potassium bromide (KBr) plates. The ^1^H NMR and ^13^C NMR spectra were determined on a Bruker Avance-400 spectrometer with DMSO-d_6_. The X-ray diffraction patterns were collected on an X-ray diffractometer (PHILIPS, PW 1510, Netherland). Scanning Electron Microscopy and Energy Dispersive X-ray analysis (MIRA3-TESCAN FESEM) was used to provide information about morphology and elemental composition. Raman spectra were recorded using a Takram N1-541 Raman spectrometer (Teksan, Tehran, Iran). Thermal analysis was carried out on a STA 503 (Bahr) instrument.

### 2.2. Synthesis of graphene oxide

Graphite powder (1 g) along with 0.5 g sodium nitrate were dissolved in 25 ml of sulphuric acid (98%) for 10 minutes and then 3 g potassium carbonate was added to the mixture while the temperature increased to 35 °C with further stirring for 30 min. Afterward, the solution was added to DI water (100 ml) and stirred for 15 minutes at 95 °C. Then, 10 ml (30% w/w) H_2_O_2_ was added to the solution. The obtained brown solution was centrifuged, washed thoroughly with HCl and DI water, and dried at 60 °C for 12 h. Finally, the dried solid was dispersed in distilled H_2_O by sonication, then centrifuged and dried again at 60 °C for 24 h.

### 2.3. Synthesis of spherical silicon dioxide nanoparticles

The mixture of EtOH (50 ml) and DI water (20 ml) was ultrasonicated for 30 minutes. Then, the solution of tetraethyl orthosilicate (3 ml) and PVP (0.1 mmol) in 5 ml ethanol was added dropwise to a mixture by vigorous stirring. Ethylenediamine (0.1 ml) as a precipitating agent was slowly added to the solution under sonication. After 30 minutes, the obtained silicon dioxide (SiO_2_) was collected and washed thoroughly with EtOH and H_2_O and then was dried at 80 °C.

### 2.4. Synthesis of ethylenediamine group@silicon dioxide

At first, refluxing silicon dioxide (1 g) with 0.5 ml 3-chloropropyl triethoxysilane (CPTES) was done in 30 ml dry toluene for 24 h. The obtained product was centrifuged and washed with toluene several times and then dried for 8 h at 120 °C. Then, 0.3 g ethylenediamine was added to a solution containing 1 g of SiO_2_@CPTES in 30 ml EtOH and refluxed for 24 h. The obtained solid was filtered and washed with water and EtOH and finally dried for 10 h under 90 °C.

### 2.5. Synthesis of ethylenediamine-functionalized SiO_2_@graphene oxide

0.04 g GO, 20 ml DI water, and 0.16 g SiO_2_@ethylenediamine were added to a round-bottom flask and dispersed under ultrasonic irradiation for 20 minutes. Then, the sonicated solution was stirred for 12 h at 85 °C and the solid material was centrifuged, washed with EtOH and DI water to remove impurities, and dried overnight at 60 °C.

### 2.6. Synthesis of ethylenediamine-functionalized SiO_2_@graphene oxide@cobalt

1 g ethylenediamine-functionalized SiO_2_@graphene oxide, 0.01 wt% cobalt(ii) chloride and 5 ml absolute EtOH were added to a flask and dispersed under ultrasound for 5 minutes to obtain a uniform dispersion after mixing. The uniform mixture was stirred at RT for 24 h. The final product was then separated and washed with EtOH and DI water and dried at RT.

### 2.7. General procedure for the synthesis of aminonaphthoquinone derivatives

To a mixture of lawsone (1 mmol) and the respective amine (1 mmol) in ethanol (5 ml), 20 wt% catalyst was added. After stirring for 10 minutes at room temperature (RT), the corresponding benzaldehyde (1 mmol) was added and stirred until the reaction was completed (monitored by TLC). The produced solid was purified by cold ethanol and water to afford the pure product.

## 3. Results and discussion

### 3.1. Characterization of catalyst

**Fig. 1:**
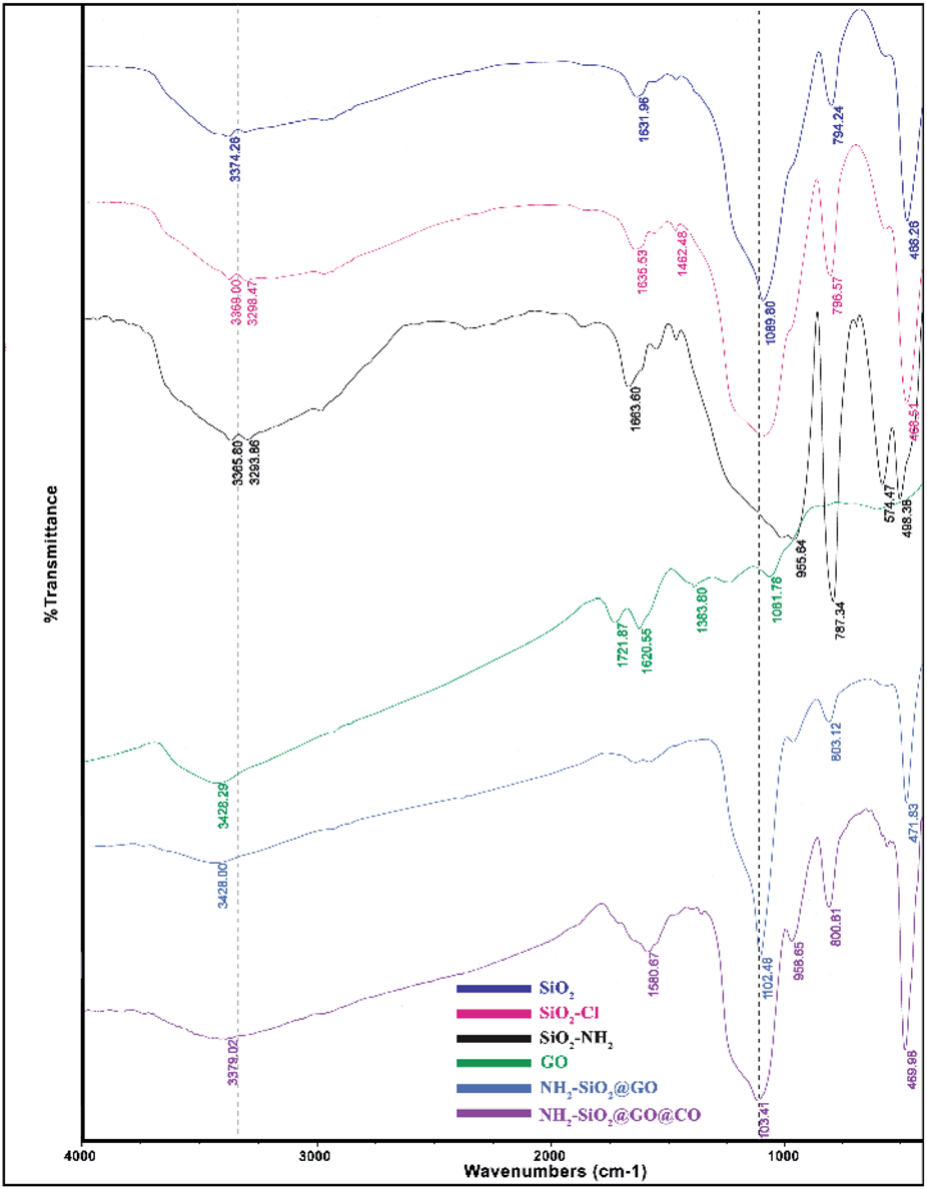
FT-IR spectra of SiO_2_, SiO_2_@Cl, SiO_2_@NH_2_, GO, f-SiO_2_@GO, and f-SiO_2_@GO@Co.

The synthesized catalyst (f-SiO_2_@GO@Co) includes the properties of functionalized SiO_2_ and GO. The FT-IR spectrum of distinct steps for the catalyst is presented in Fig. 1. Graphene oxide showed peaks at 3398 cm^−1^, 1721 cm^−1^, 1383, and 1060 cm^−1^, which were attributed to the O–H group stretching vibrations, carbonyl stretch for carboxylic acid, C–O stretching, and epoxy group vibrations, respectively. For SiO_2_, the absorption peaks at 798, 1089 cm^−1^, and 3370 cm^−1^ were attributed to asymmetric vibrations of the Si–O–Si bonds and O–H groups. In SiO_2_@Cl and SiO_2_@ethylenediamine spectra, the bands at around 950 cm^−1^ were related to ethoxy stretching vibrations. After grafting functionalized SiO_2_ onto graphene oxide, the band at 1102 cm^−1^ confirmed the successful formation of f-SiO_2_@GO@Co.

**Fig. 2:**
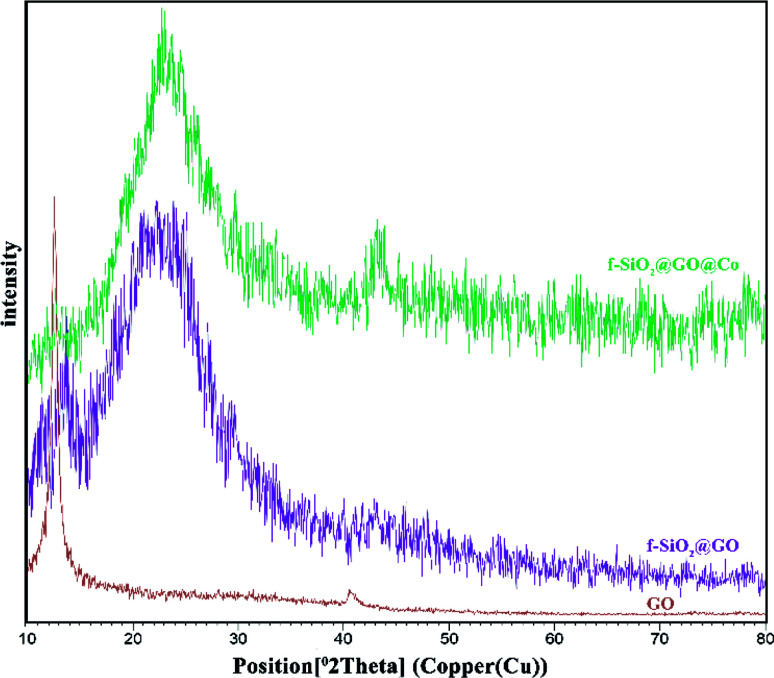
XRD patterns of GO, f-SiO_2_@GO, and f-SiO_2_@GO@Co.

The XRD pattern for graphene oxide sheets showed a strong peak at about 2*θ* = 12°, which revealed the exfoliation of GO and intercalation of water molecules in the graphite structure such that oxygen functional groups result between graphite layers.^39^ After the functionalization of graphene oxide, the removal of many oxygen-containing functional groups on the surface of graphene oxide cause the intensity of the peak at 2*θ* = 12° to decrease. Also, the broad peak at 2*θ* = 25° and 2*θ* = 44° corresponded to the amorphous silica and Co, which suggests the successful synthesis of the desired catalyst (Fig. 2).

**Fig. 3:**
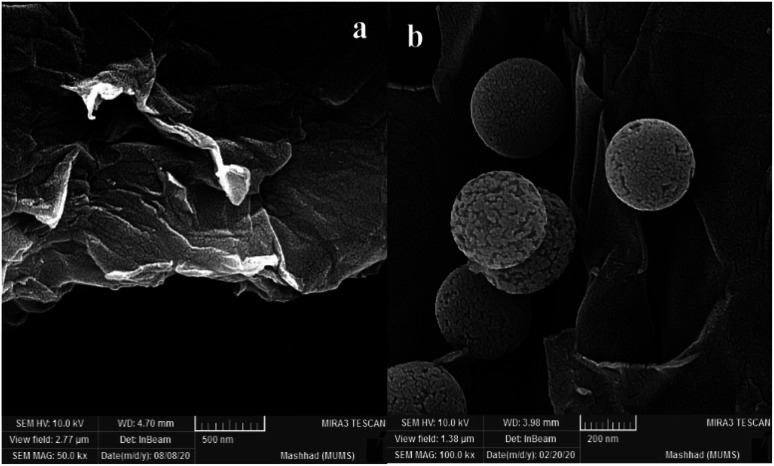
FE-SEM images of GO (a) f-SiO_2_@GO@Co (b).

The SEM analysis for graphene oxide (Fig. 3a) shows the layered structure with crumpled morphology at the sheet edges. Functionalized graphene oxide (Fig. 3b) exhibits the distribution of functionalized spherical silica nanoparticles on the surface of graphene oxide sheets.

**Fig. 4:**
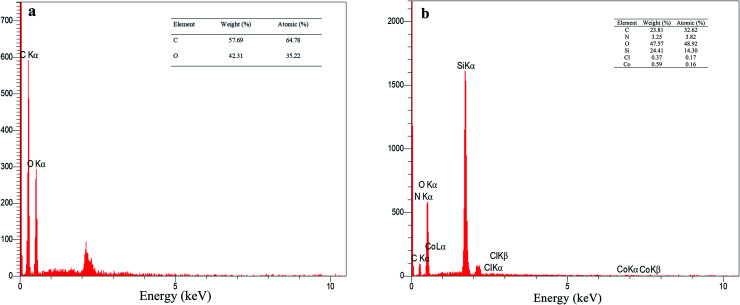
EDS spectra of GO (a) and f-SiO_2_@GO@Co (b).

The EDX analysis for graphene oxide and f-SiO_2_@GO@Co catalyst are presented in Fig. 4. According to the data for graphene oxide EDX (Fig. 4a), the carbon and oxygen contents for graphene oxide (atomic percentage) were 64.78% and 35.22%, respectively, and the ratio of carbon to oxygen was C/O = 1.83. The atomic percentage of C, N, O, Si, Cl, Co was 32.62%, 3.82%, 48.92%, 14.30%, 0.17%, 0.16%, respectively. In the elemental analysis of f-SiO_2_@GO@Co, SiO_2_ moiety in the catalyst structure reduced the carbon amount (32.62%) and increased the oxygen amount (48.92%), which resulted in a carbon to oxygen ratio of C/O = 0.66.^40^

**Fig. 5:**
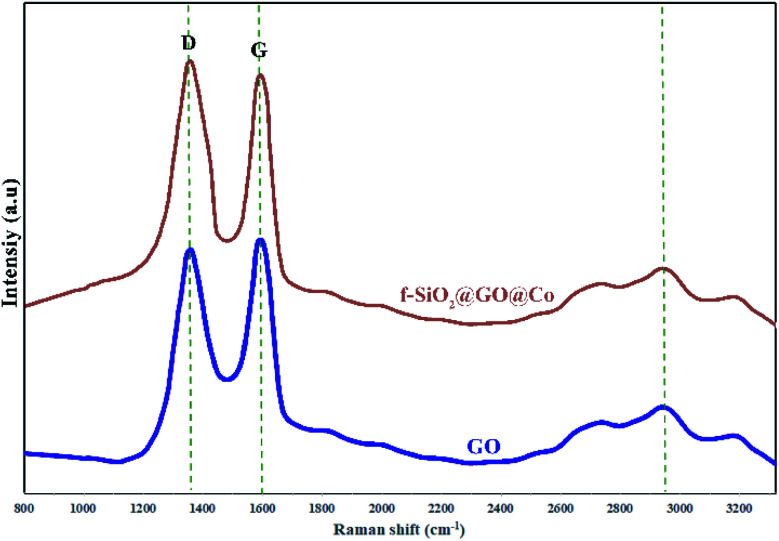
The Raman spectra for GO and f-SiO_2_@GO@Co.

The Raman spectra were used for the identification of structural properties of GO and GO@f-SiO_2_@Co (Fig. 5). Two characteristic peaks for graphene oxide at 1595 cm^−1^ (in-plane vibrations of sp^2^ bonded carbon) and 1338 cm^−1^ (out of plane vibrations) were related to G and D bands, respectively. The Raman spectrum for functionalized graphene oxide showed similar peaks, while *I*_D_/*I*_G_ ratio increased compared to *I*_D_/*I*_G_ of graphene oxide, indicating the formation of more sp^3^ carbon in f-SiO_2_@GO@Co from the grafting of f-SiO_2_ on graphene oxide.^41,42^

**Fig. 6:**
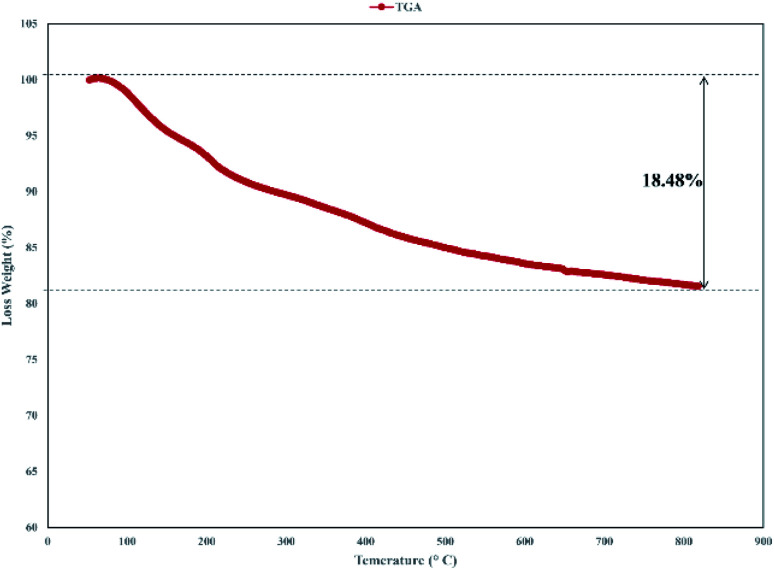
The TGA analysis for f-SiO_2_@GO@Co.

Thermogravimetric analysis (TGA) of the catalyst was carried out and the result is shown in Fig. 6. The TGA spectrum of graphene oxide shows that it is unstable, with weight loss even below 100 °C corresponding to the removal of absorbed water.^43^ In TGA of the catalyst, the primary loss occurred at around 200 °C, which was attributed to the pyrolysis of the functional groups containing oxygen.^44^ Decomposition of the catalyst after 230 °C, that continues to 800 °C indicated the thermal stability of the catalyst and related to the decomposition of functional groups in GO.^45^

To show the efficiency of the catalyst, we tested its potential in the model reaction. For this purpose, different parameters such as solvents, variety of catalysts, time, and the amount of the desired catalyst were evaluated on the model reaction.

In order to display the superiority of the catalyst, different catalysts were compared to a model reaction (4b). First, the reaction was performed without the catalyst and no product was formed after 20 h (Table 1, entry 1). Then, different nanoparticles were used and the obtained yield was checked. The nanoparticles resulted in low yield. Functionalized SiO_2_ was then used on Fe_3_O_4_ and graphene oxide support. The obtained result showed that graphene oxide was a better support with more activity. Finally, f-SiO_2_@GO and f-SiO_2_@GO@Co were compared. f-SiO_2_@GO@Co resulted to have more acidic sites and better catalytic efficiency with the maximum yield (98%) in a minimum time (Table 1, entry 7).

**Table 1:** The results of different catalysts on the model reaction^a^

Entry	Catalyst	Time^b^	Yield^c^ (%)
1	—	20 h	—
2	NiO NPs	7 h	40%
3	SiO_2_ NPs	3 h	60%
4	NiO@SiO_2_ NPs	3 h	52%
5	f-SiO_2_@Fe_3_O_4_	1 h	70%
6	f-SiO_2_@GO	30 min	85%
**7**	**f-SiO**_**2**_ **@GO@Co**	**5 min**	**98%**

a Reaction conditions: 2-hydroxynaphthoquinone (1 mmol), 4-nitrobenzaldehyde (1 mmol), 3-nitro aniline (1 mmol), different catalysts (20 mg), RT. stirring.

b Reaction progress monitored by TLC.

c Isolated yield.

In continuation, different protic (ethanol, water, and ethanol/water) and aprotic (acetonitrile, THF, and toluene) solvents were tested in the presence of f-SiO_2_@GO@Co (20%) as a catalyst. The results indicated that the reaction in the presence of protic solvents was faster than that in the presence of aprotic solvents and the final yields ranged between 87% and 98%. The best protic solvent was ethanol, which resulted in 98% yield of appropriate product in minimum reaction time (Table 2, entry 2).

**Table 2:** Evaluation of different solvents on the model reaction^a^

Entry	Solvent	Time^b^	Yield^c^ (%)
1	THF	150 min	42%
**2**	**Ethanol**	**5 min**	**98%**
3	Water	50 min	87%
4	Ethanol/water	50 min	92%
5	Toluene	200 min	30%
6	Acetonitrile	90 min	42%

a Reaction conditions: 2-hydroxynaphthoquinone (1 mmol), 4-nitrobenzaldehyde (1 mmol), 3-nitroaniline (1 mmol), f-SiO_2_@GO@Co (20%), different solvents, RT. stirring.

b Reaction progress monitored by TLC.

c Isolated yield.

Then, the effect of 10 and 20% (w/w) f-SiO_2_ loading on the GO surface was evaluated. The result indicated that 20% (w/w) f-SiO_2_ on the surface of GO increases the catalyst's efficiency. Then, the impact of f-SiO_2_@GO@Co amount in the reaction was evaluated (Table 3, entries 1–5). The obtained result exhibited that the catalyst loading of 20 wt% increased the yield up to 98% (Table 3, entry 4) while loading higher than 20 wt% did not have any impact on improving the yield. After finding the best conditions for the reaction, aminonaphthoquinone derivatives in the presence of the catalyst (20%) at room temperature in ethanol were synthesized (Table 4).

**Table 3:** Evaluation of catalyst loading for upgrading the yield of the reaction^a^

Entry	Catalyst loading	Time^b^	Yield^c^ (%)
1	5	2 h	62%
2	10	90 min	74%
3	15	45 min	85%
**4**	**20**	**5 min**	**98%**
5	30	5 min	98%

a Reaction conditions: 2-hydroxynaphthoquinone (1 mmol), 4-nitrobenzaldehyde (1 mmol), 3-nitro aniline (1 mmol), f-SiO_2_@GO@Co different amounts, RT. stirring.

b Reaction progress monitored by TLC.

c Isolated yield.

**Table 4:** Synthesis of aminonaphthoquinone derivatives by f-SiO_2_@GO@Co as a catalyst

Entry	Aldehyde	Amine	Product	Time (min)	Yield (%)
1	4-Cl	4-NO_2_	4a	5	98%
2	4-NO_2_	3-NO_2_	4b	5	98%
3	3-OH	4-NO_2_	4c	7	96%
4	3-NO_2_	3-NO_2_	4d	7	97%
5	2-OH	4-NO_2_	4e	8	96%
6	4-CH_3_	4-NO_2_	4f	7	96%
7	Ph	4-NO_2_	4g	6	96%
8	2-OH-5-Br	4-NO_2_	4h	6	96%
9	4-OCH_3_	4-NO_2_	4i	7	96%
10	4-N(CH_3_)_2_	4-NO_2_	4j	7	96%
11	3,4-Dimethoxy	4-NO_2_	4k	7	95%
12	2-NO_2_	4-NO_2_	4l	5	96%

### 3.2. Reusability of the catalyst

**Fig. 7:**
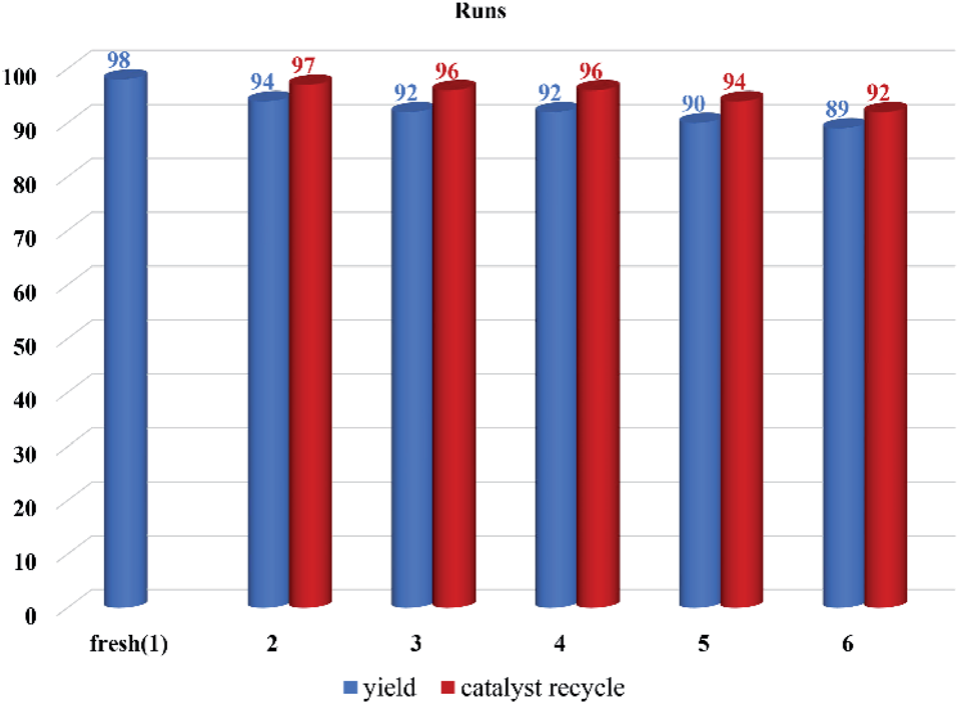
Recycling results of f-SiO_2_@GO@Co on the model reaction.

The reusability of the catalyst was examined by conducting the recyclability test in the model reaction (4b). The catalyst was separated at the end of the reaction by centrifugation and washed with ethyl acetate to remove impurities. The catalyst was used six times with a great average recycling value (95%). The results showed that the catalytic activity decreased from 98% in the first run (fresh) to 89% after the completion of the sixth run (Fig. 7).

### 3.3. Analysis and characterization of the synthesized compound

**Fig. 8:**
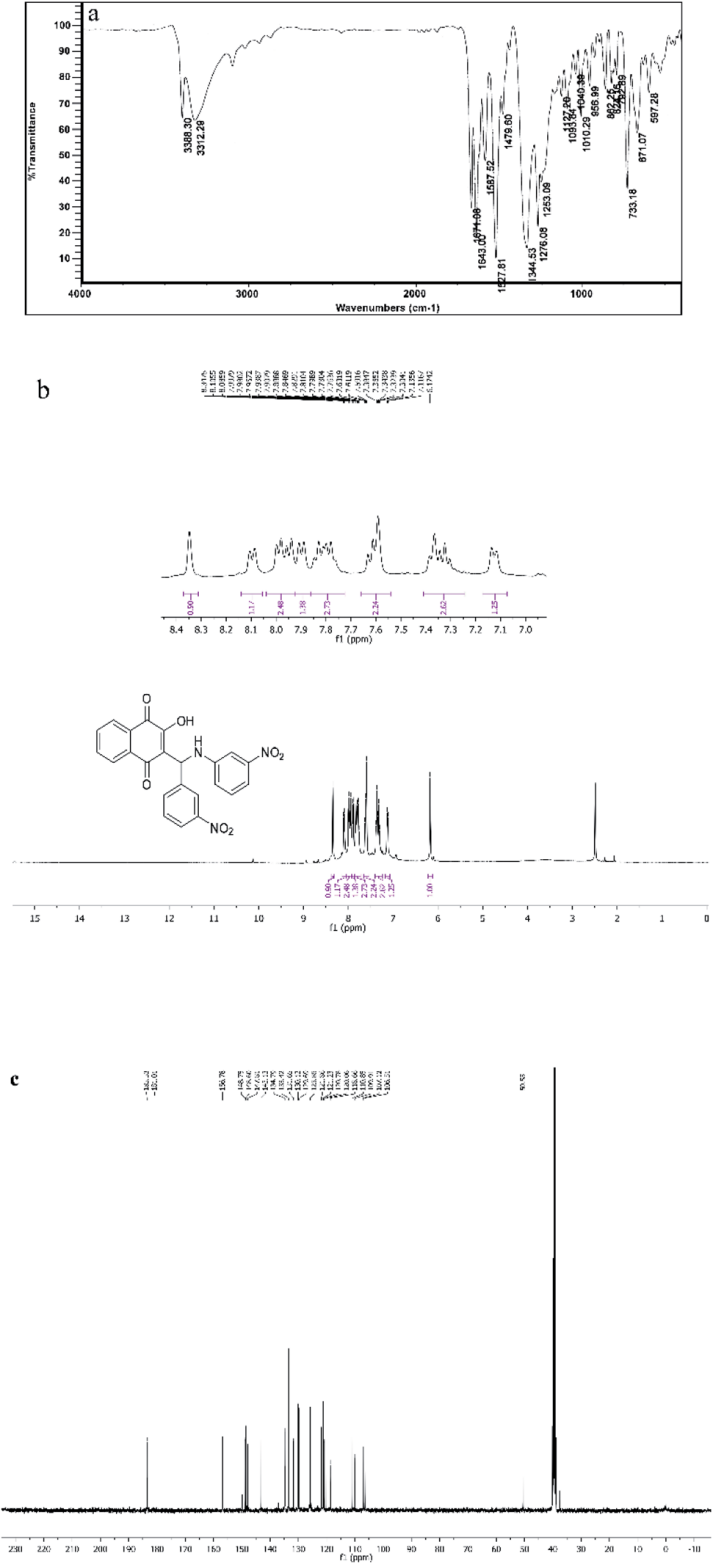
FT-IR, ^1^HNMR, ^13^CNMR spectra of compound 4d.

All the synthesized compounds (4a–l) were characterized with different methods such as FT-IR and ^1^H NMR and ^13^C NMR spectroscopy. The IR spectrum of the selected compound (4d) shows peaks at 3388 and 3312 cm^−1^, which were related to OH and NH symmetric stretching vibrations. The band at 1642 cm^−1^ was for C=O stretching vibration (Fig. 8a). The ^1^H NMR spectrum of compound 4d exhibited a single characteristic peak at *δ* = 6.17 ppm for the CH group. The D_2_O exchangeable protons of amine and hydroxyl were observed in the region of 7–8.30 ppm (Fig. 8b). The ^13^CNMR spectrum of compound 4d showed a prominent peak at around 50.55 ppm, which was related to the CH group and the peaks at 185 ppm and 181 ppm correspond to the carbonyl groups (Fig. 8c).

### 3.4. Proposed mechanism

**Scheme 2:**
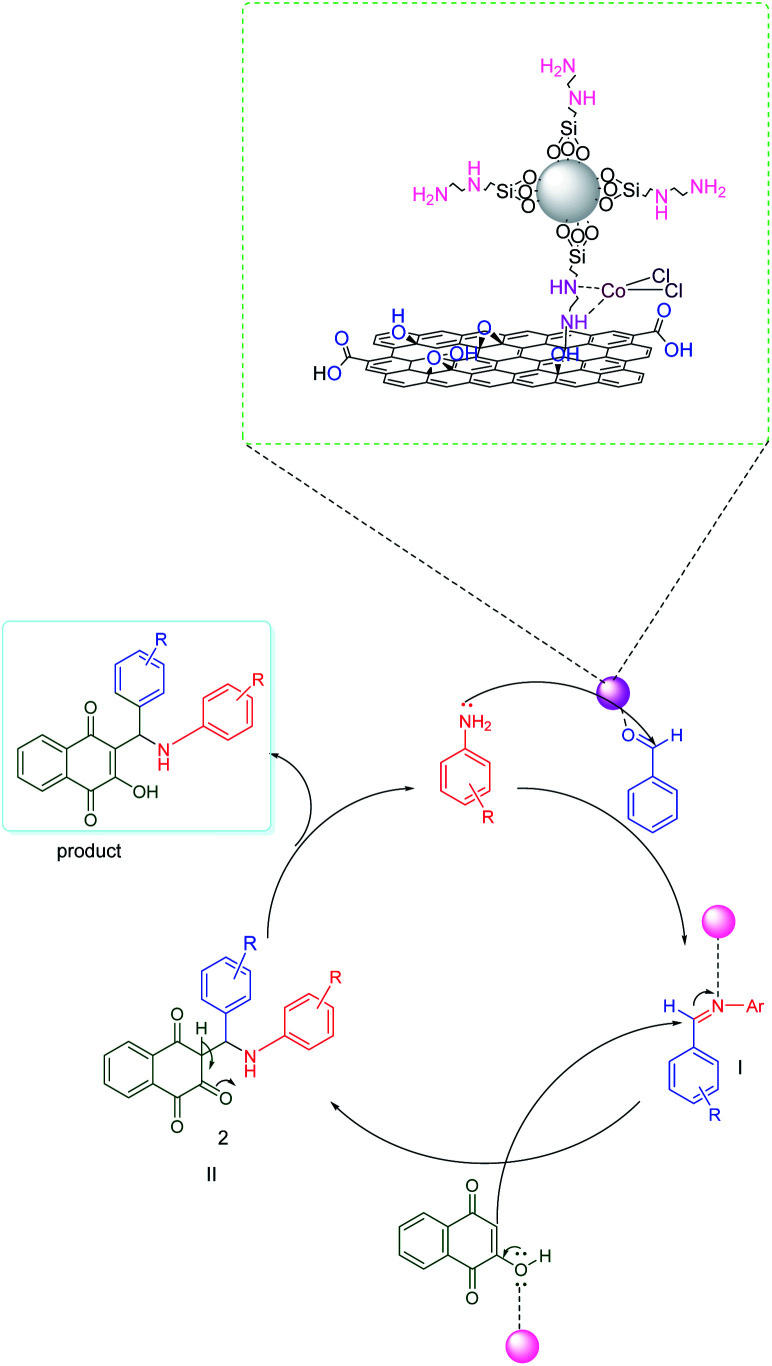
A proposed mechanism for the synthesis of aminonaphthoquinone derivatives.

According to the proposed mechanism presented in Scheme 2, aromatic amines react with activated benzaldehyde in the presence of the catalyst through nucleophilic attack to form activated imine as an intermediate(i). Then, the intermediate(i) is attacked by 2-hydroxynaphthalene-1,4-dione through intermolecular H-atom transfer and nucleophilic addition, which gives intermediate(ii) and will undergo tautomerization to form the final products.

### 3.5. Comparison of the performance of f-SiO_2_@GO@Co with some previously reported heterogenous catalysts

Comparative tests were performed to check the ability of f-SiO_2_@Go@Co as opposed to other previously reported catalysts in the literature to synthesise aminonaphthoquinone. The results revealed that f-SiO_2_@Go@Co had a better catalytic performance than the other catalysts in terms of yield and production time (Table 5, entry 5).

**Table 5:** Comparison of the catalytic potential of f-SiO_2_@GO@Co with previously reported catalysts for the synthesis of aminonaphthoquinone

Entry	Catalyst	Solvent	Temperature	Time	Yield (maximum)	Ref.
1	Montmorillonite-k10	Ethanol	r.t.	8 h	93	46
2	PPA	H_2_O	Ambient	6 h	87.9	47
3	InCl_3_	H_2_O	Reflux	6.5 h	91	48
4	γ-Fe_2_O_3_/SiO_2_-propyl-NH-AMAM-SO_3_H	Solvent-free	Ambient condition	3 h	93	49
**5**	**f-SiO2@GO@Co**	**Ethanol**	**r.t.**	**5 min**	**98%**	**This work**

## 4. Conclusion

GO@f-SiO_2_@Co is a heterogenous catalyst synthesized with spherical silica particles grafted on the surface of graphene oxide with the help of ethylenediamine ligand and coordination with Co(ii). The activity of the catalyst for the synthesis of aminonaphthoquinones has been assessed. The results showed that the catalyst with high catalytic activity gave excellent yield under mild conditions in a short reaction time.

## Conflicts of interest

There are no conflicts to declare.

## Supplementary Material

RA-011-D1RA01790J-s001

## References

[cit1] Novoselov K. S., Geim A. K., Morozov S. V., Jiang D., Zhang Y., Dubonos S. V., Grigorieva I. V., Firsov A. A. (2004). Science.

[cit2] Wang X., Zhi L., Müllen K. (2008). Nano Lett..

[cit3] Ramanathan T., Abdala A. A., Stankovich S., Dikin D. A., Herrera-Alonso M., Piner R. D., Adamson D. H., Schniepp H. C., Chen X., Ruoff R. S., Nguyen S. T., Aksay I. A., Prud'Homme R. K., Brinson L. C. (2008). Nat. Nanotechnol..

[cit4] Yang Q., Pan X., Clarke K., Li K. (2012). Ind. Eng. Chem. Res..

[cit5] Sur U. K. (2012). Int. J. Electrochem..

[cit6] Zhu Y., Murali S., Cai W., Li X., Suk J. W., Potts J. R., Ruoff R. S. (2010). Adv. Mater..

[cit7] Bonaccorso F., Colombo L., Yu G., Stoller M., Tozzini V., Ferrari A. C., Ruoff R. S., Pellegrini V. (2015). Science.

[cit8] Ray S. C. , Application and Uses of Graphene Oxide and Reduced Graphene Oxide, Elsevier Inc., 2015

[cit9] Aday B., Pamuk H., Kaya M., Sen F. (2016). J. Nanosci. Nanotechnol..

[cit10] Bozkurt S., Tosun B., Sen B., Akocak S., Savk A., Ebeoğlugil M. F., Sen F. (2017). Anal. Chim. Acta.

[cit11] Lavin-Lopez M. D. P., Romero A., Garrido J., Sanchez-Silva L., Valverde J. L. (2016). Ind. Eng. Chem. Res..

[cit12] Hajian R., Fung K., Chou P. P., Wang S. W., Balderston K. A. (2019). Mater. Matters.

[cit13] Chen D., Feng H., Li J. (2012). Chem. Rev..

[cit14] Chaurasia S. R., Dange R., Bhanage B. M. (2020). Catal. Commun..

[cit15] Dandia A., Bansal S., Sharma R., Rathore K. S., Parewa V. (2018). RSC Adv..

[cit16] Vijay Kumar A., Rama Rao K. (2011). Tetrahedron Lett..

[cit17] Li Z., Wang R., Young R. J., Deng L., Yang F., Hao L., Jiao W., Liu W. (2013). Polymer.

[cit18] Jiang T., Kuila T., Kim N. H., Ku B. C., Lee J. H. (2013). Compos. Sci. Technol..

[cit19] Wan Y. J., Gong L. X., Tang L. C., Bin Wu L., Jiang J. X. (2014). Composites, Part A.

[cit20] Vennerberg D., Rueger Z., Kessler M. R. (2014). Polymer.

[cit21] Wang X., Xing W., Zhang P., Song L., Yang H., Hu Y. (2012). Compos. Sci. Technol..

[cit22] Haeri S. Z., Ramezanzadeh B., Asghari M. (2017). J. Colloid Interface Sci..

[cit23] Shi X., Nguyen T. A., Suo Z., Liu Y., Avci R. (2009). Surf. Coat. Technol..

[cit24] Wang T., Ge H., Zhang K. (2018). J. Alloys Compd..

[cit25] Banerjee A. N. (2018). Interface Focus.

[cit26] Georgakilas V., Perman J. A., Tucek J., Zboril R. (2015). Chem. Rev..

[cit27] Alvand M., Shemirani F. (2017). Microchim. Acta.

[cit28] Seifvand N., Kowsari E. (2015). RSC Adv..

[cit29] Lumby R. J. R., Joensuu P. M., Lam H. W. (2007). Org. Lett..

[cit30] Wang J., Feng K., Zhang H. H., Chen B., Li Z. J., Meng Q. Y., Zhang L. P., Tung C. H., Wu L. Z. (2014). Beilstein J. Nanotechnol..

[cit31] Khurana J. M., Lumb A., Chaudhary A., Nand B. (2013). Synth. Commun..

[cit32] Feitosa Dos Santos A., Ferraz P. A. L., Ventura Pinto A., Pinto M. D. C. F. R., Goulart M. O. F., Sant'Ana A. E. G. (2000). Int. J. Parasitol..

[cit33] Chen J., Huang Y. W., Liu G., Afrasiabi Z., Sinn E., Padhye S., Ma Y. (2004). Toxicol. Appl. Pharmacol..

[cit34] Gafner S., Wolfender J. L., Nianga M., Stoeckli-Evans H., Hostettmann K. (1996). Phytochemistry.

[cit35] Kuder J. E., Wychick D., Miller R. L., Walker M. S. (1974). J. Phys. Chem..

[cit36] Esteves-Souza A., Araújo Lúcio K., da Cunha A. S., da Cunha Pinto A., da Silva Lima E. L., Camara C. A., Domingues Vargas M., Gattass C. R. (2008). Oncol. Rep..

[cit37] Silva T. M. S., Camara C. A., Barbosa T. P., Soares A. Z., Da Cunha L. C., Pinto A. C., Vargas M. D. (2005). Bioorg. Med. Chem..

[cit38] Tandon V. K., Yadav D. B., Singh R. V., Chaturvedi A. K., Shukla P. K. (2005). Bioorg. Med. Chem. Lett..

[cit39] Siddiqui S., Siddiqui Z. N. (2020). Nanoscale Adv..

[cit40] Yau X. H., Low F. W., Khe C. S., Lai C. W., Tiong S. K., Amin N. (2020). PLoS One.

[cit41] Calizo I., Balandin A. A., Bao W., Miao F., Lau C. N. (2007). Nano Lett..

[cit42] Kudin K. N., Ozbas B., Schniepp H. C., Prud'homme R. K., Aksay I. A., Car R. (2008). Nano Lett..

[cit43] Mikhaylov P. A., Vinogradov M. I., Levin I. S., Shandryuk G. A. (2019). Mater. Sci. Eng..

[cit44] Stankovich S., Dikin D. A., Piner R. D., Kohlhaas K. A., Kleinhammes A., Jia Y., Wu Y., Nguyen S. B. T., Ruoff R. S. (2007). Carbon.

[cit45] Huang Y., Yan W., Xu Y., Huang L., Chen Y. (2016). Chem. Synth. Appl. Graphene Carbon Mater..

[cit46] Jayashree S., Shivashankar K. (2018). Synth. Commun..

[cit47] Liu D., Zhou S., Gao J. (2014). Synth. Commun..

[cit48] Dabiri M., Tisseh Z. N., Bazgir A. (2011). Dyes Pigm..

[cit49] Mollazehi F., Shaterian H. R. (2018). Appl. Organomet. Chem..

