# Supplementary material for: Design, synthesis, and catalytic performance of modified graphene oxide based on a cobalt complex as a heterogenous catalyst for the preparation of aminonaphthoquinone derivatives
Source: RSC Adv. 2021 May 11;11(28):17108–15. doi: 10.1039/d1ra01790j (PMC9032551; doi:10.1039/d1ra01790j)
Supplement: RA-011-D1RA01790J-s001 [file RA-011-D1RA01790J-s001.pdf]

Electronic Supplementary Material (ESI) for RSC Advances.
This journal is © The Royal Society of Chemistry 2021

Design, synthesis, and catalytic performance of modified graphene oxide based on cobalt complex as a heterogenous catalyst for the preparation of aminonaphthoquinone derivatives

Mahnaz Mirheidari [a] and Javad Safaei-Ghomi [a,*]

Department of Organic Chemistry, Faculty of Chemistry, University of Kashan, Kashan, P.O. Box 87317-51167, I.R. Iran.

**Corresponding Author Tel.: +98 31 55912385; fax: +98 31 55552935; *E-mail: safaei@kashanu.ac.ir*

General procedure for the synthesis of aminonaphthoquinone derivatives

To a mixture of lawsone (1mmol) and the respective amine (1mmol) in ethanol (5ml), 20%wt catalyst was added. After stirring for 10 minutes at room temperature, the corresponding benzaldehyde (1mmol) was added and stirred until the reaction was completed (monitored by TLC). Then, the solid was purified by cold ethanol and water to afford the pure product.

Spectral data

2-((4-Chlorophenyl)((4-nitrophenyl)amino)methyl)-3-hydroxynaphthalene-1,4-dione(4a): Orange solid, mp. 125-128 °C, IR (KBr): υ =3365,1674,1640,1597; ^{1}HNMR (DMSO ,400 MHz) δ = 8.04 – 7.93 (m, 5H), 7.88 – 7.79 (m, 2H), 7.72 (m,1H), 7.47-7.42 (d, J = 8.2 Hz, 2H), 7.40-7.35 (d, J = 8.2 Hz, 2H), 6.80-6.73 (d, J = 8Hz, 2H), 6.10 (s, 1H).

2-((4-Nitrophenyl)((3-nitrophenyl)amino)methyl)-3-hydroxynaphthalene-1,4-dione (4b): Orange solid, mp.170-172 °C, IR (KBr): 3381, 3258, 1674, 1636; ^{1}HNMR (DMSO, 400 MHz); δ= 8.20-8.15 (d, J = 8.4 Hz, 2H), 8.02-7.99 (d, J = 7.8 Hz, 2H), 7.96-7.92 (d, J = 7.8 Hz, 2H), 7.86 – 7.79 (m,2H), 7.74-7.71 (d, J = 8 Hz, 2H), 7.56 (S, 1H), 7.39 – 7.31 (m, 2H), 7.11 (m, 1H), 6.15 (S, 1H); ^{13}C NMR (DMSO, 400 MHz):δ= 183.42, 181.02, 156.64, 153.49, 138.84, 136.38, 134.81, 133.43, 131.63, 131.47, 131.15,130.06, 129.34, 128.53, 128.14, 126.38,126.10, 125.86, 120.70, 112.33, 50.41.

2-((3-Hydroxyphenyl)(4-nitrophenyl)amino)methyl)-3-hydroxynaphthalene-1,4-dione(4c): Yellow solid, mp.122-124 oC, IR (KBr): 3393, 1674, 1594; 1HNMR (DMSO ,400 MHz): δ= 9.33 (S, 1H), 8.03 – 7.92 (m, 5H), 7.89 – 7.77 (m, 2H), 7.64(m, 1H), 7.09 (t, J = 8 Hz, 1H), 6.87-6.82 (d, J = 5.9 Hz, 2H), 6.80-6.72 (m, 2H), 6.61(m, 1H), 6.05 (S, 1H).

2-((3-Nitrophenyl)(3-nitrophenyl)amino)methyl)-3-hydroxynaphthalene-1,4-dione (4d): Orange solid, mp.180-183 ºC, IR (KBr): 3388, 3312, 1671, 1643; ^{1}HNMR (DMSO,400 MHz): δ= 8.35 (s, 1H), 8.10 (d, J = 8Hz, 1H), 8.01-7.93 (m, 2H), 7.89 (d, J = 7.6 Hz, 1H), 7.85 – 7.74 (m, 3H), 7.64 – 7.58 (t, J = 8 Hz, 2H), 7.38 – 7.30 (m,3H), 7.15-7.12 (d, J = 7.6 Hz, 1H), 6.17 (S, 1H); ^{13}C NMR (DMSO, 400 MHz):δ=183.53, 181.01, 156.78, 148.75, 148.60, 147.81, 143.13,134.79, 133.42, 131.65, 130.12, 129.69, 125.86, 121.86,121.23, 120, 78, 120.06, 118.66, 110.85, 109.91, 107.12, 106.51, 50.55

2-((2-hydroxyphenyl)(4-nitrophenyl)amino)methyl)-3-hydroxynaphthalene-1,4-dione(4e): Orange solid, mp. 246-248 °C, IR (KBr): 3349, 3063, 1686, 1641; ^{1}HNMR (DMSO, 400 MHz): 8.13 – 8.08 (m, 2H), 8.07– 7.99 (m, 4H), 7.92 – 7.87 (m, 2H), 7.67 (m, 1H), 7.51(m, 1H), 7.46-7.42 (m, 2H), 7.28 (m, 1H), 7.06 – 6.96 (m, 2H), 6.25 (d, J = 8 Hz, 1H).

2-((4-methylphenyl)(4-nitrophenyl)amino)methyl)-3-hydroxynaphthalene-1,4-dione (4f): Yellow solid, mp. 160-162 °C, IR (KBr): 3386, 3355, 1645, 1598; ^{1}HNMR (DMSO, 400 MHz): δ= 8.03 – 7.93 (m, 5H), 7.86 – 7.76 (m, 2H), 7.67 (m, 1H), 7.35-7.28 (d, J = 7.6 Hz, 2H), 7.14-7.09 (d, J = 7.6 Hz, 2H), 6.81 – 6.72 (m, 2H), 6.08 (S, 1H), 2.25 (S, 3H).

2-((phenyl)(4-nitrophenyl)amino)methyl)-3-hydroxynaphthalene-1,4-dione (4g): Orange solid, mp. 180-183 °C, IR (KBr): 3342, 1644, 1598; ^{1}HNMR (DMSO, 400 MHz):δ= 8.02– 7.95 (m, 5H), 7.86 – 7.77 (m, 2H), 7.71 (S, 1H),7.48- 7.45 (d, J = 7.4 Hz, 2H), 7.34-7.29 (t, J = 7.4 Hz, 2H), 7.23 (t, J = 7.0 Hz,1H), 6.82 – 6.75 (m, 2H), 6.13 (S,1H).

2-((2-hydroxy-5-bromophenyl)(4-nitrophenyl)amino)methyl)-3-hydroxynaphthalene-1,4-dione (4h): Orange solid, mp. 282-285 °C, IR (KBr):3367, 1687,1641, 1594; ^{1}HNMR (DMSO, 400 MHz): δ= 8.12– 7.98 (m, 5H), 7.92 – 7.86 (m, 2H), 7.69 – 7.60 (m, 4H), 7.47-7.42 (d, J = 8.4 Hz, 1H), 7.06 – 7.00 (m, 2H), 6.28-6.22 (d, J = 9.2 Hz, 1H); ^{13}C NMR (400 MHz, DMSO-d6): δ= 182.74, 177.82, 152.18, 150.68, 148.04, 136.56, 134.94, 134.28, 132.49, 131.44, 131.26, 130.49, 126.25, 126.19, 125.95, 124.30, 119.72, 118.23, 117.17, 111.70, 50.53.

2-((4-methoxyphenyl)(4-nitrophenyl)amino)methyl)-3-hydroxynaphthalene-1,4-dione(4i): Orange solid, 170-172 °C, IR (KBr): 3386, 3337, 1642, 1597; ^{1}HNMR (DMSO, 400 MHz): δ= 8.01-7.93 (m, 5H), 7.86 – 7.75 (m, 3H), 7.71-7.68 (s,1H), 7.36 (d, J = 8.4 Hz, 1H), 6.87 (d, J = 8.4Hz, 1H), 6.79 – 6.71 (m,2H), 6.58(d, J = 8.8 Hz, 1H), 6.06 (S, 1H), 3.70 (S, 3H).

2-((N,N-dimethylphenyl)(4-nitrophenyl)amino)methyl)-3-hydroxynaphthalene-1,4-dione (4j): Yellow solid, 182-185 °C, IR (KBr): 3440, 1672, 1642, 1597; ^{1}HNMR (DMSO, 400 MHz): δ= 7.96 – 7.90 (t, J = 7.1Hz, 5H), 7.82 – 7.70 (m, 5H), 7.15-7.10 (d, J = 7.2 Hz, 2H), 6.92 – 6.84 (m, 2H), 6.15 (s, 1H), 2.94 (s, 6H).

2-((3,4-methoxyphenyl)(4-nitrophenyl)amino)methyl)-3-hydroxynaphthalene-1,4-dione(4k): Yellow solid, 175-178 °C, IR (KBr): 3359, 1677, 1645, 1598; ^{1}HNMR (DMSO, 400 MHz): δ= 8.02-7.90 (m,5H), 7.85-7.76 (m, 2H), 7.68 (s, 1H), 7.07 (s, 1H), 6.98-6.93 (d, J = 6.4 Hz ,1H), 6.89-6.83(t, J = 8.1Hz, 2H), 6.78-6.73 (m,1H), 6.05 (s, 1H), 3.71(s, 6H).

2-((2-Nitrophenyl)(4-nitrophenyl)amino)methyl)-3-hydroxynaphthalene-1,4-dione(4l):orange solid, 181-183 °C, IR (KBr): 3352, 1644, 1598; ^{1}HNMR (DMSO, 400 MHz): δ= 8.03 – 7.98 (m, 4H), 7.96-7.92(m, 2H), 7.89 – 7.79 (m,3H), 7.74(m,1H), 7.69-7.62(m, 1H), 7.57 – 7.51 (m,1H), 6.72 – 6.67 (m,2H), 6.64 (s, 1H).

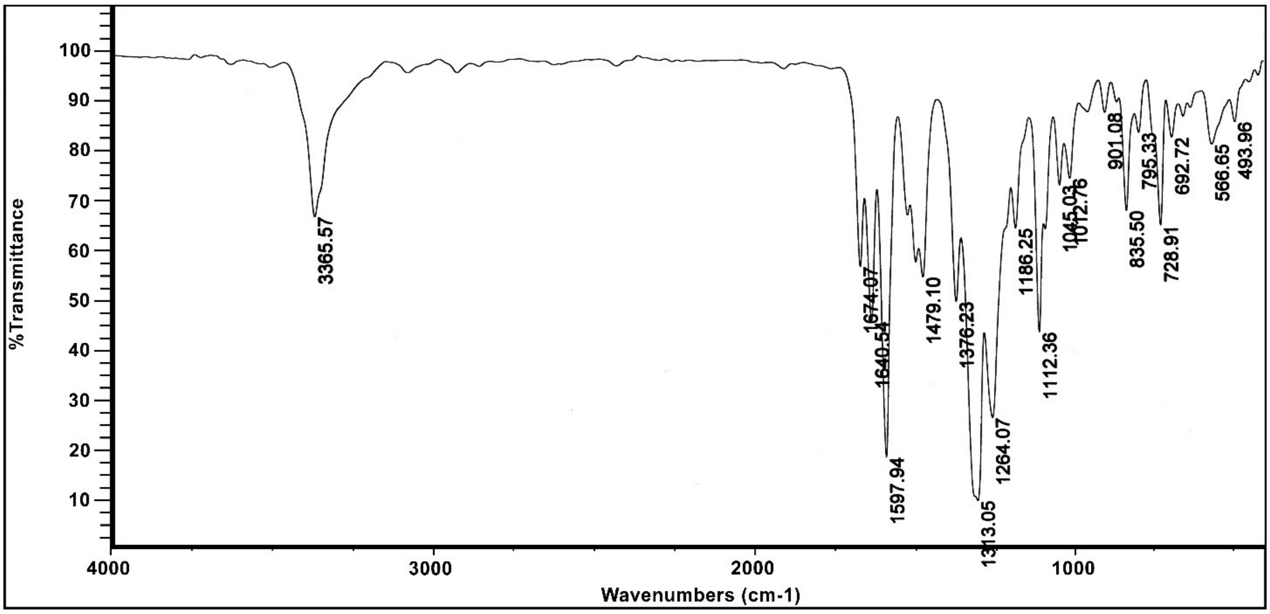

Figure S1. FT-IR spectrum of compound 4a

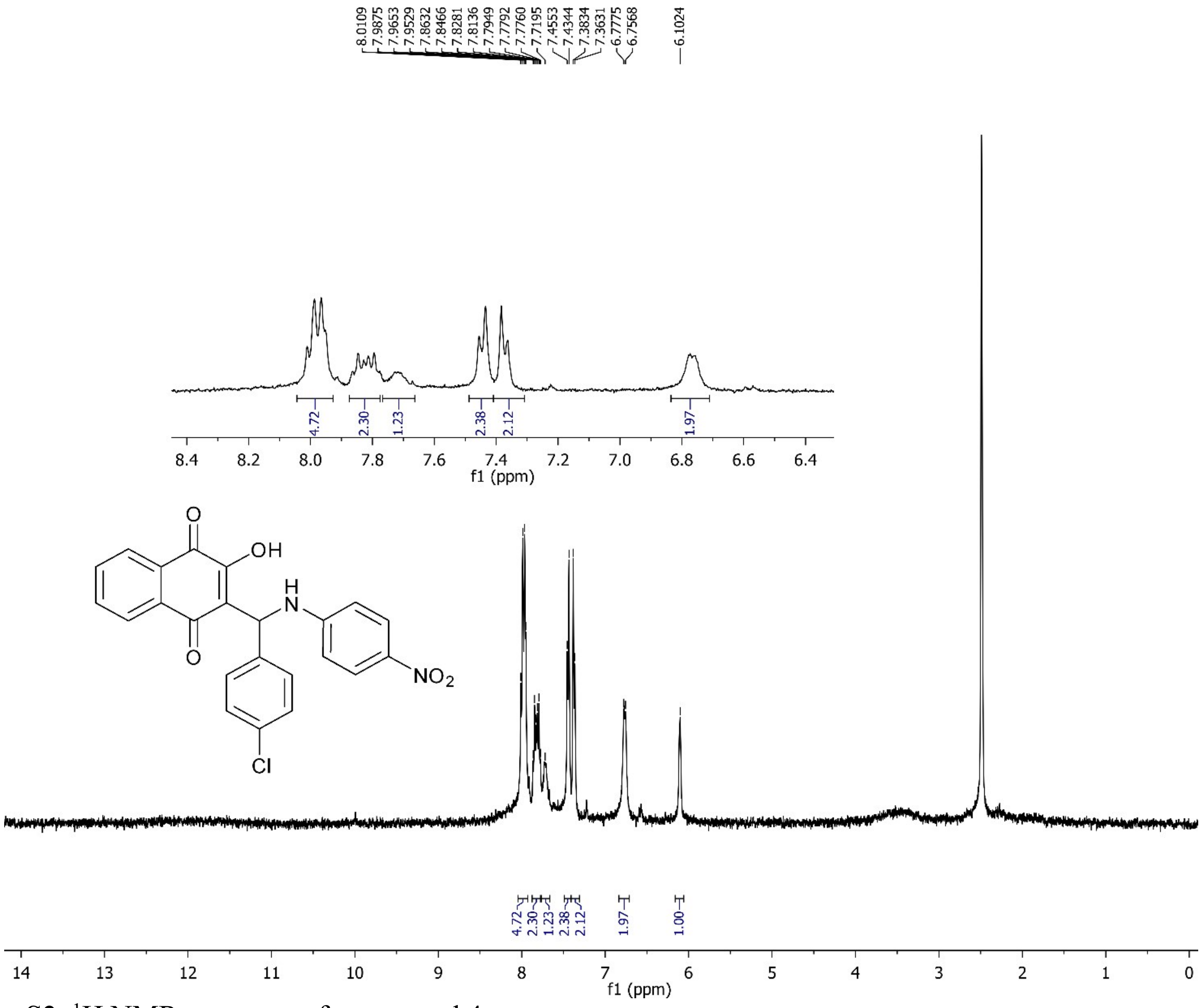

Figure S2. ^{1}H NMR spectrum of compound 4a.

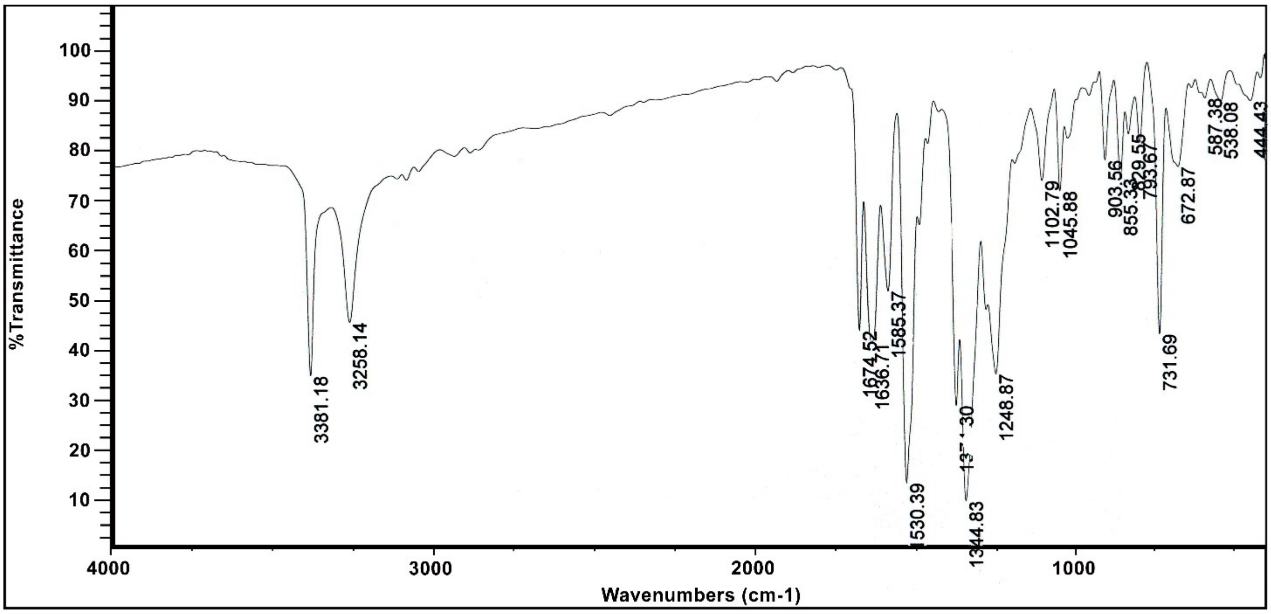

Figure S3. FT-IR spectrum of compound 4b.

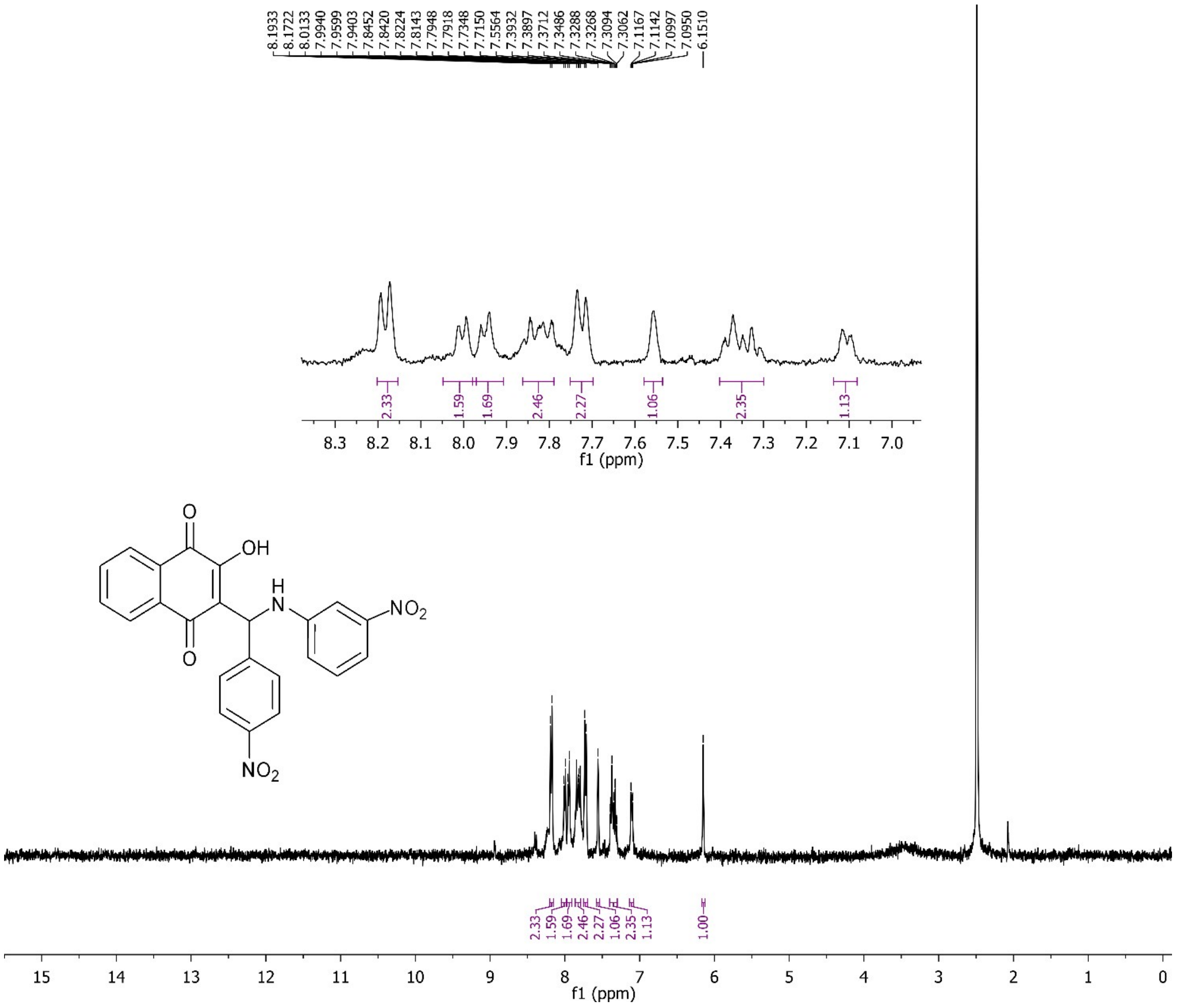

Figure S4. ^{1}H NMR spectrum of compound 4b.

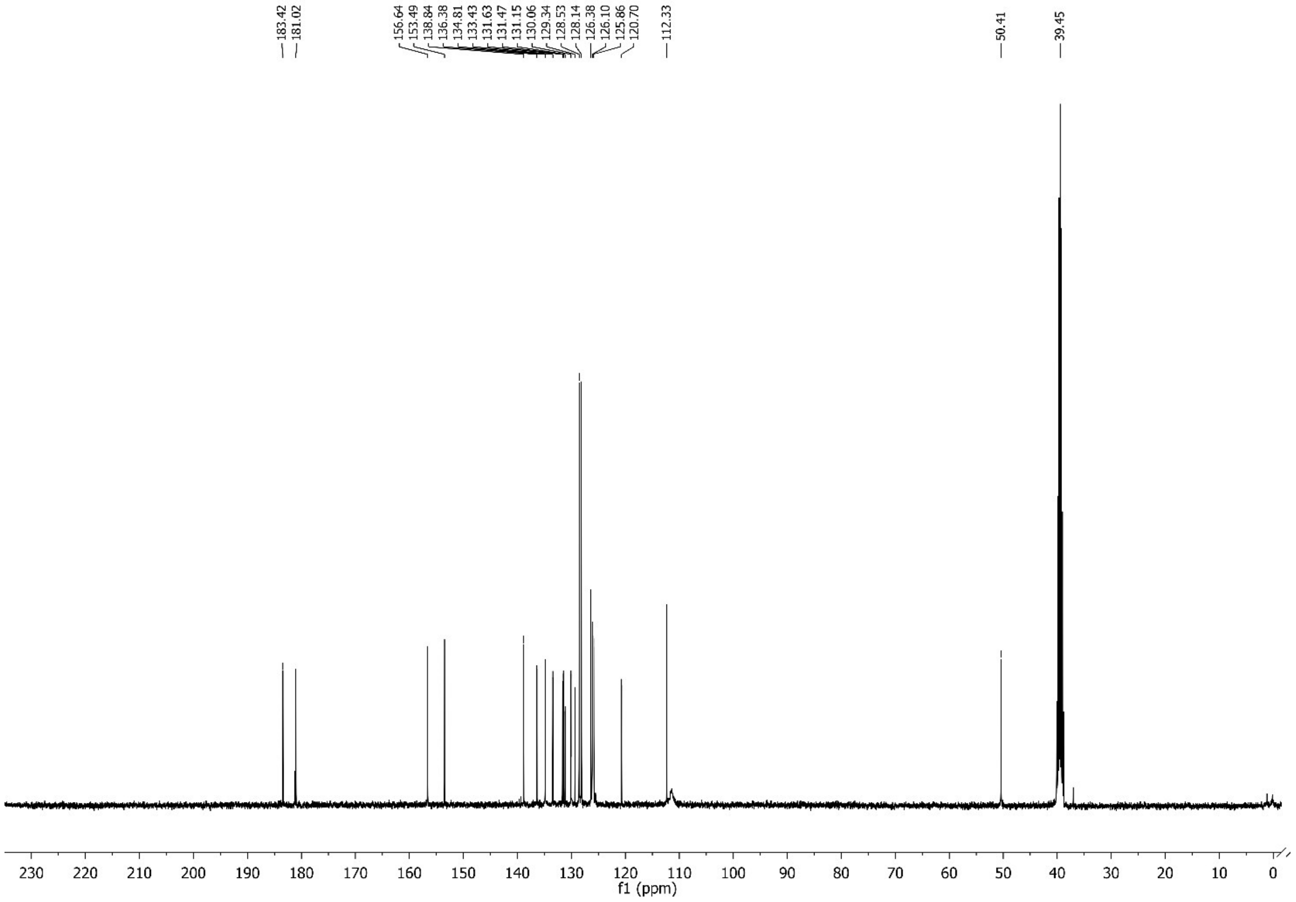

Figure S5. ^{13}C NMR spectrum of compound 4b.

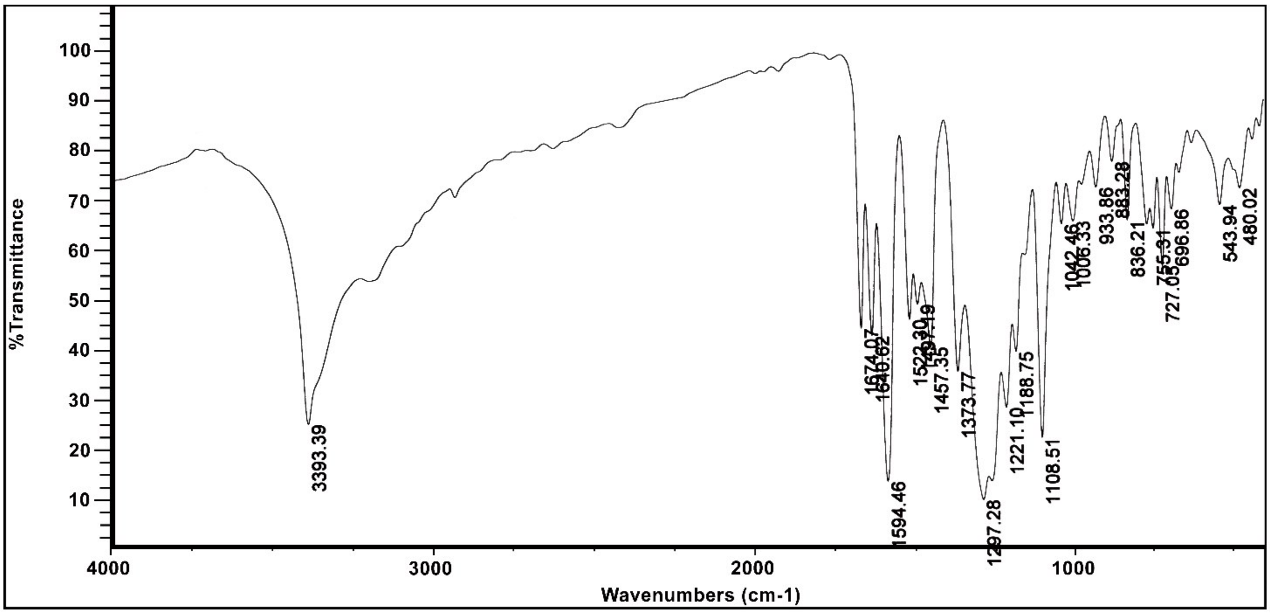

Figure S6. FT-IR spectrum of compound 4c.

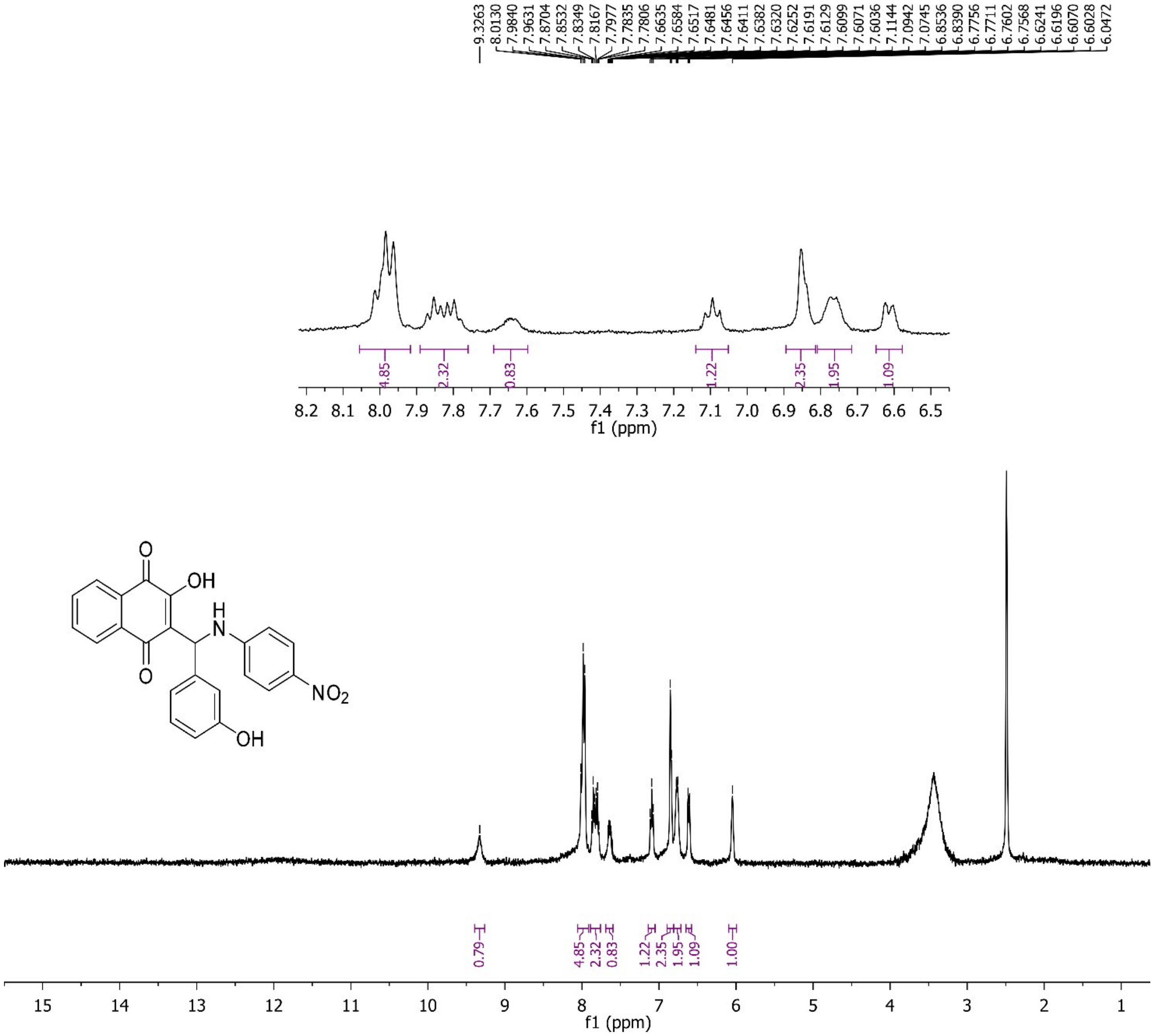

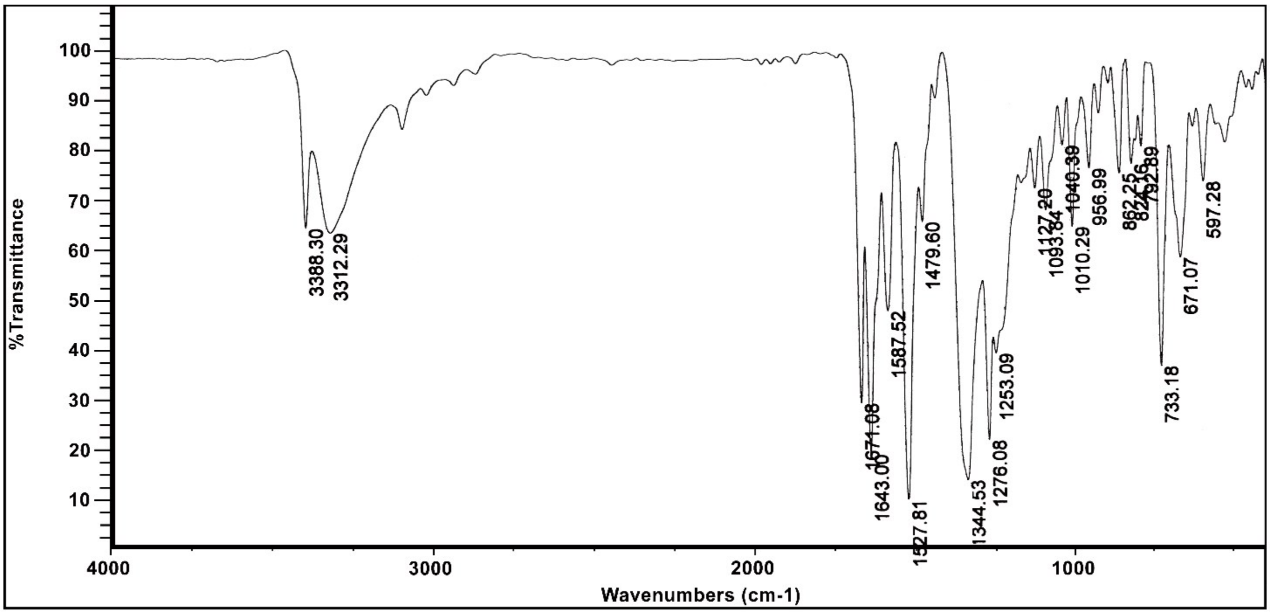

Figure S7. ^{1}H NMR spectrum of compound 4c.

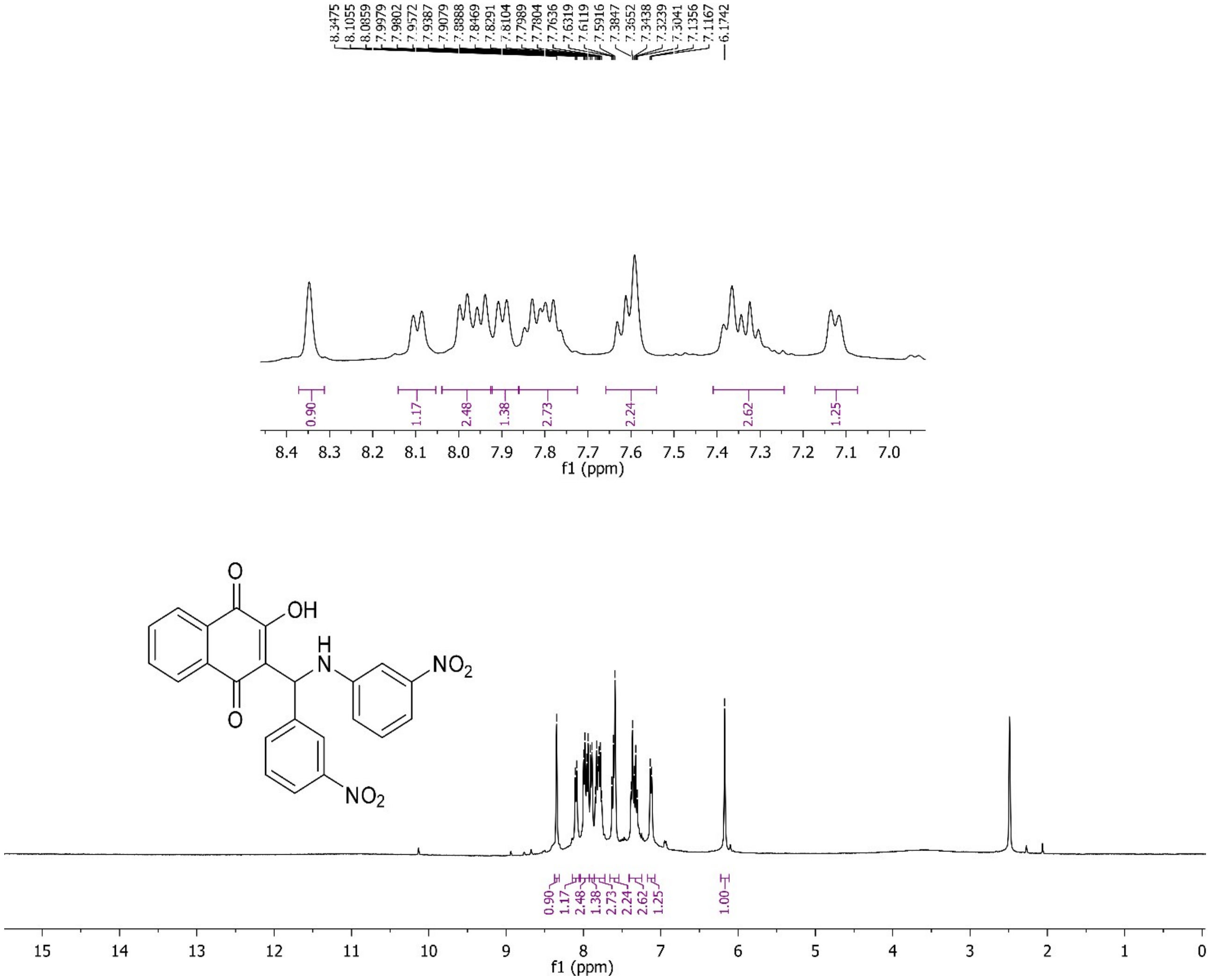

Figure S8. FT-IR spectrum of compound 4d.

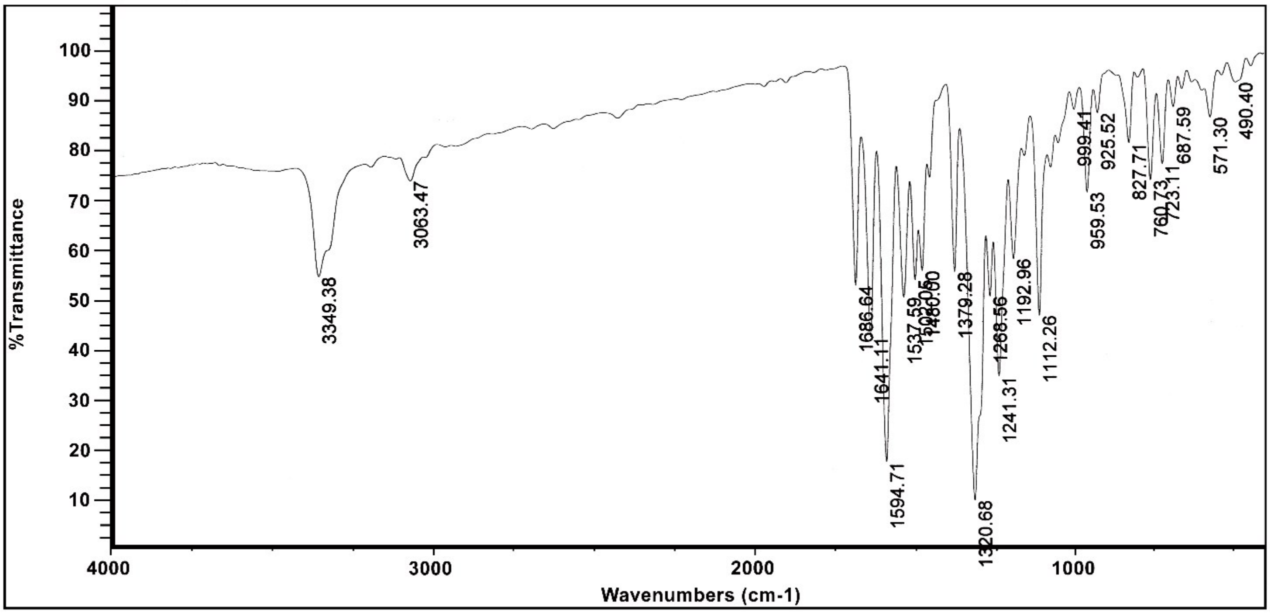

Figure S9. ^{1}H NMR spectrum of compound 4d.

Figure S10. FT-IR spectrum of compound 4e.

Figure S11. ^{1}H NMR spectrum of compound 4e.

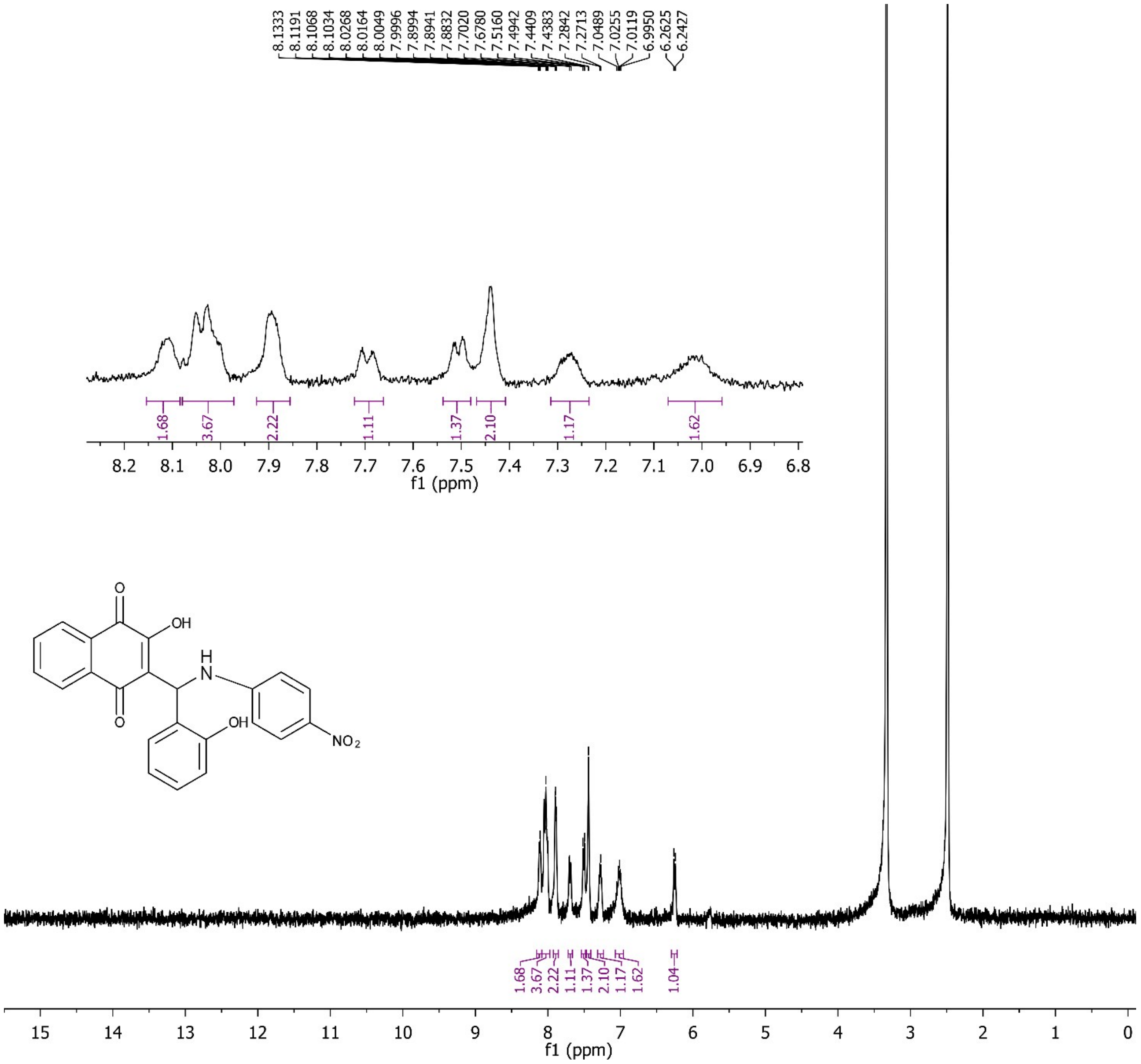

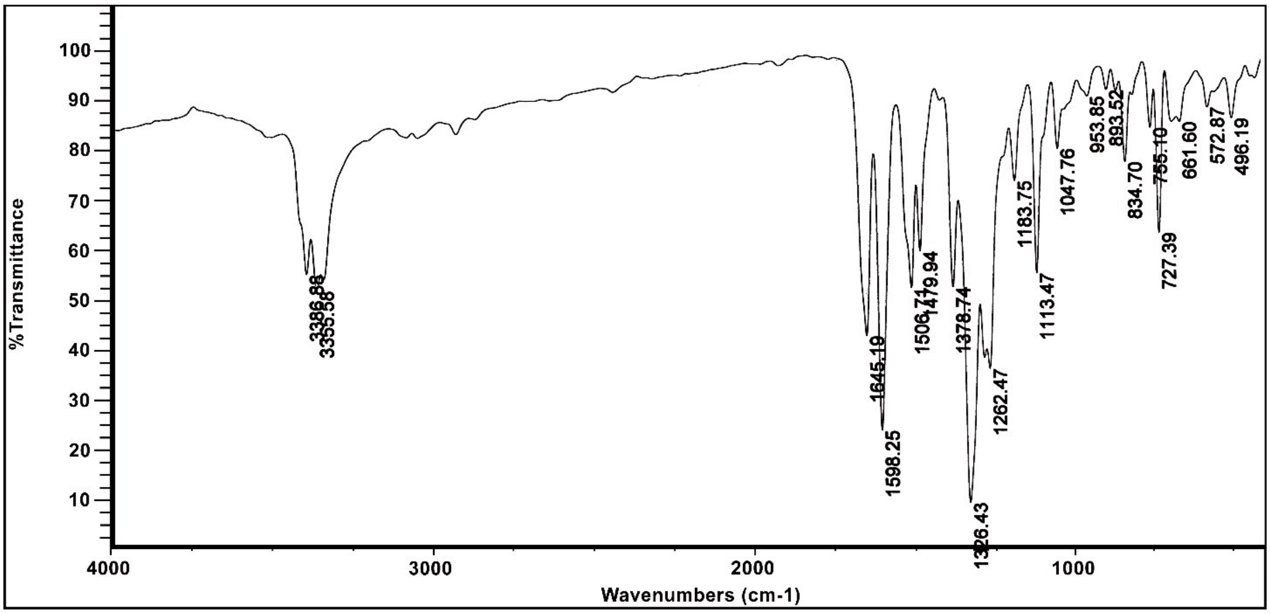

Figure S12. ^{1}H NMR spectrum of compound 4f.

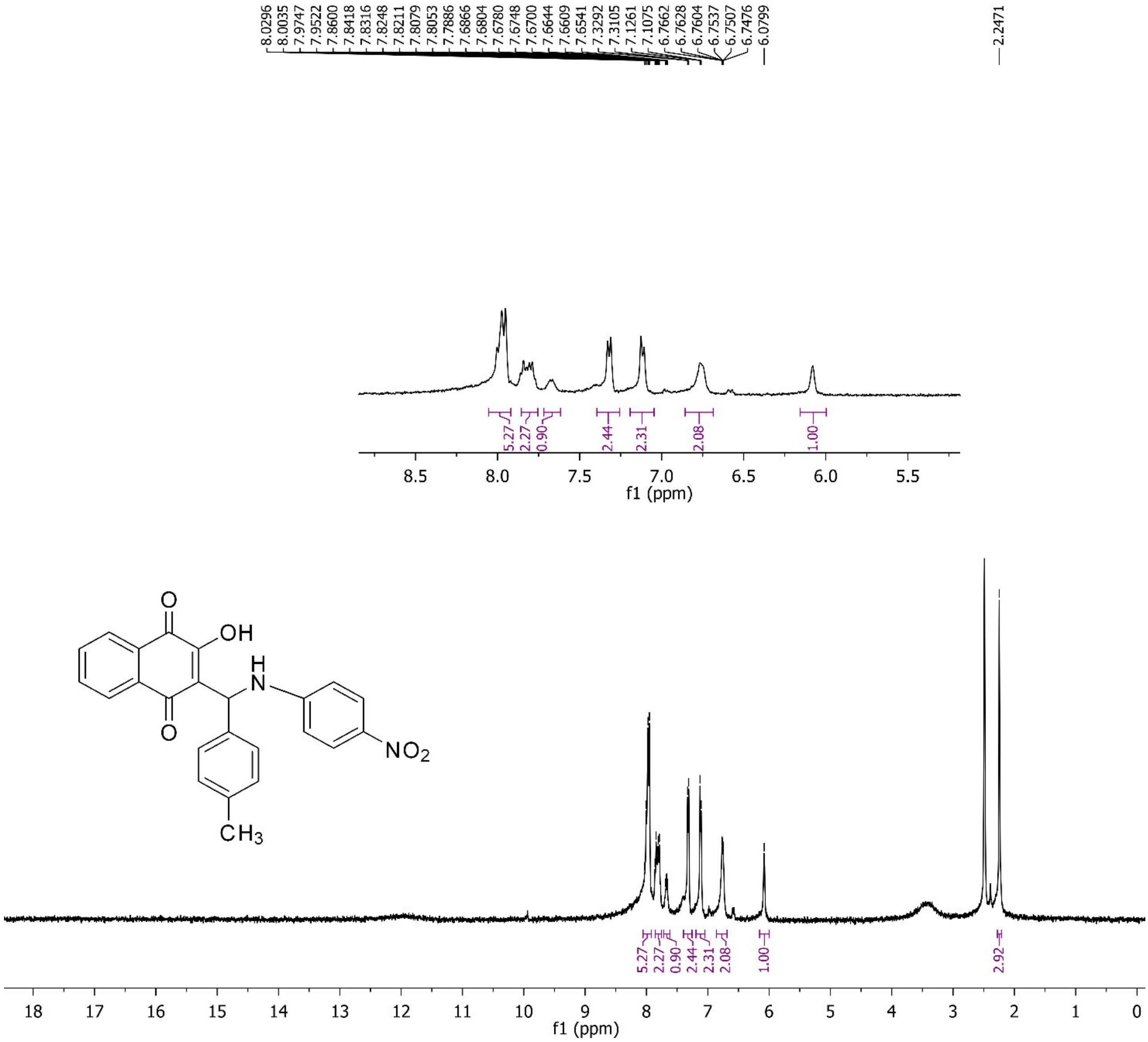

Figure S13. ^{1}H NMR spectrum of compound 4f.

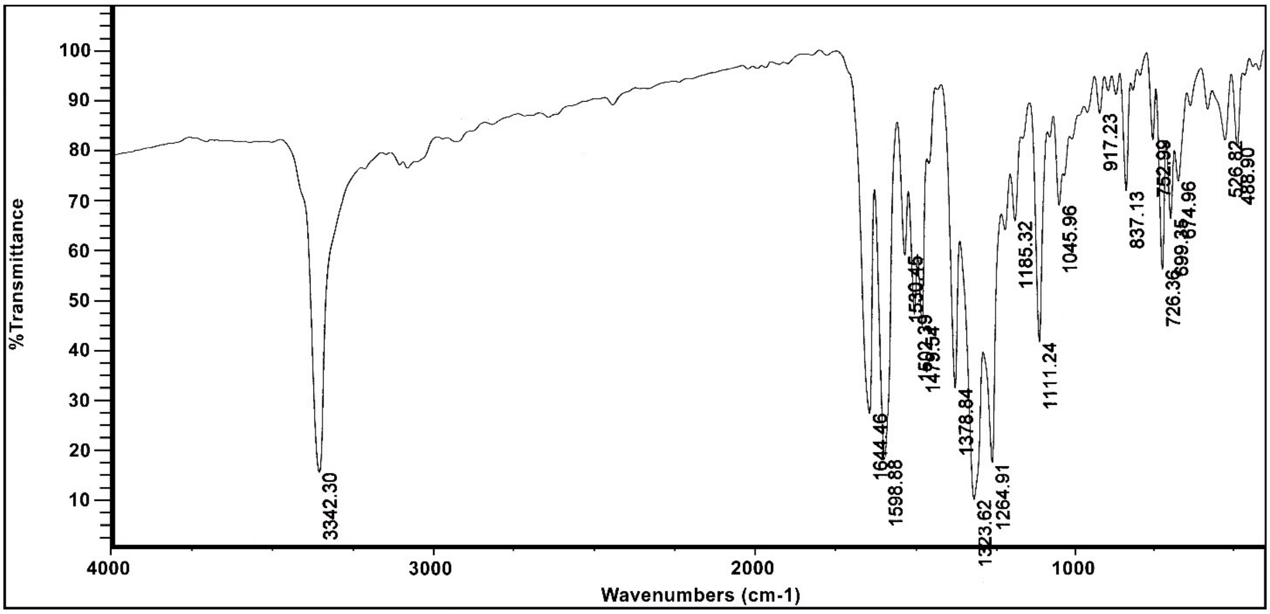

Figure S14. FT-IR spectrum of compound 4g.

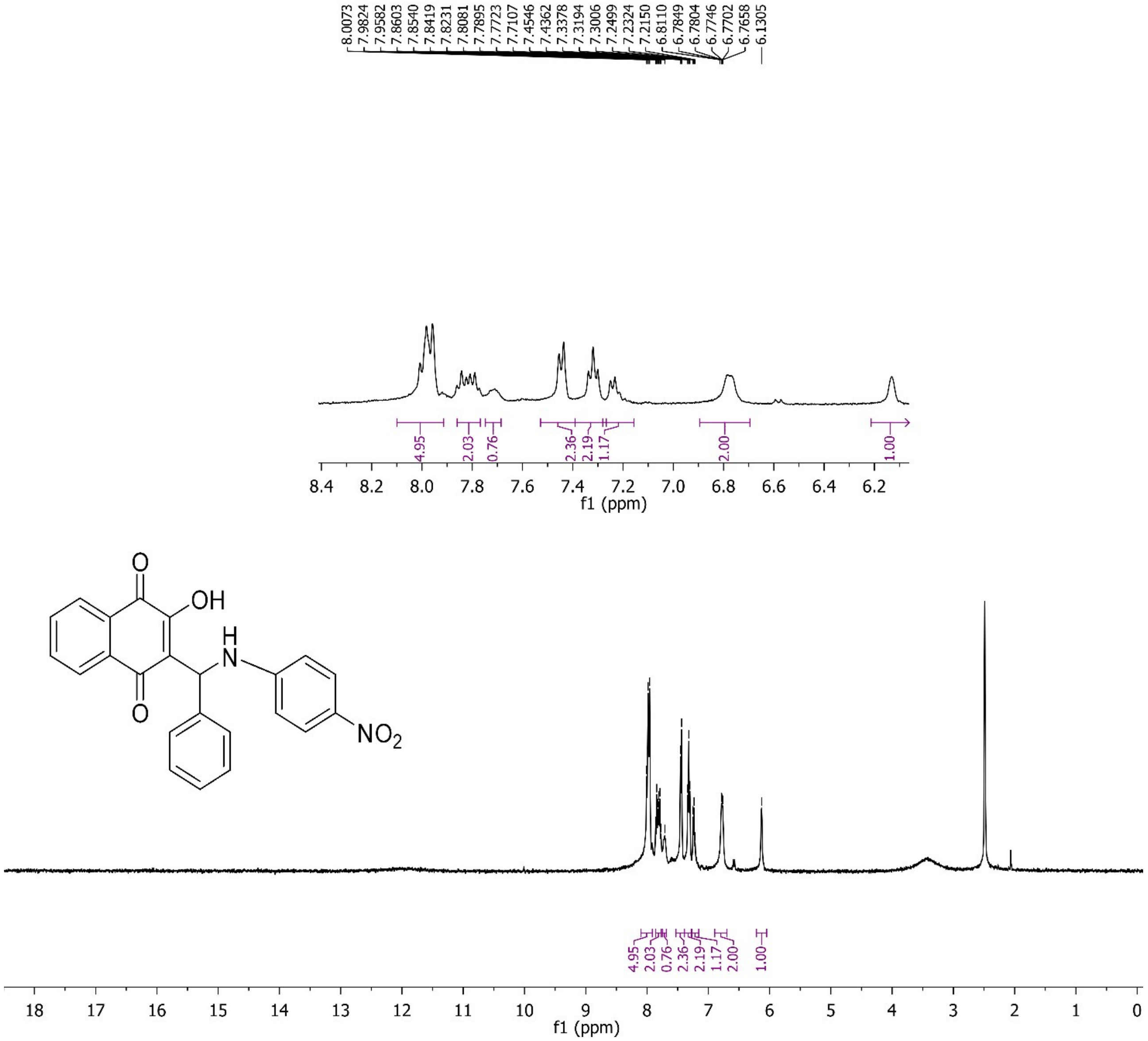

Figure S15. ^{1}H NMR spectrum of compound 4g.

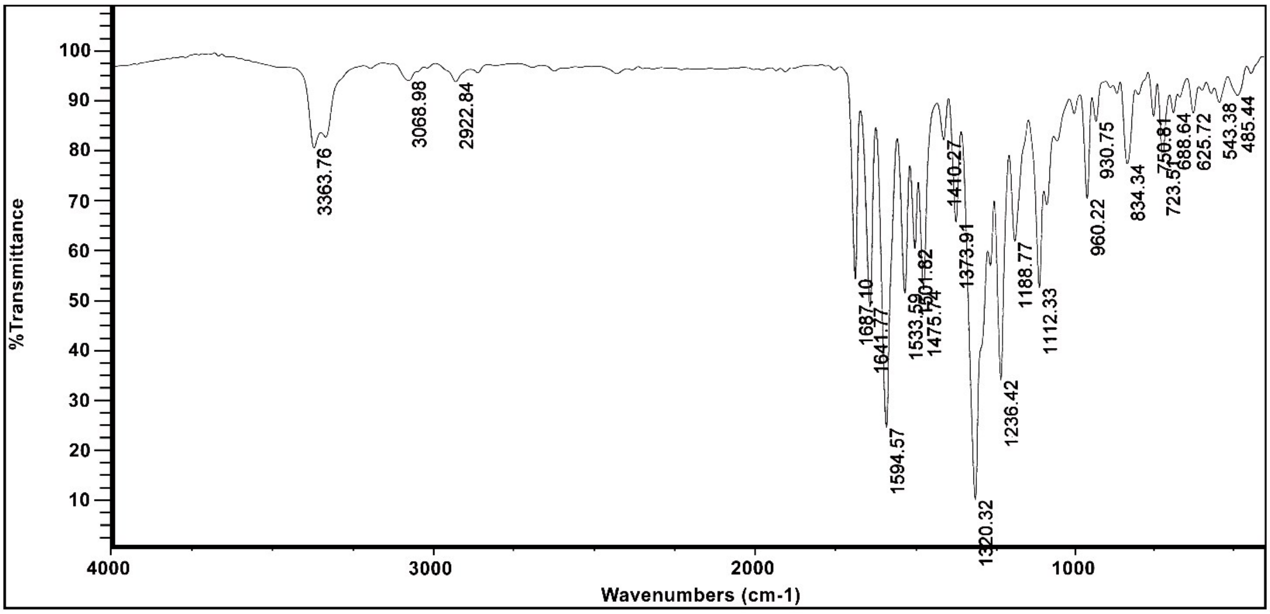

Figure S16. FT-IR spectrum of compound 4h.

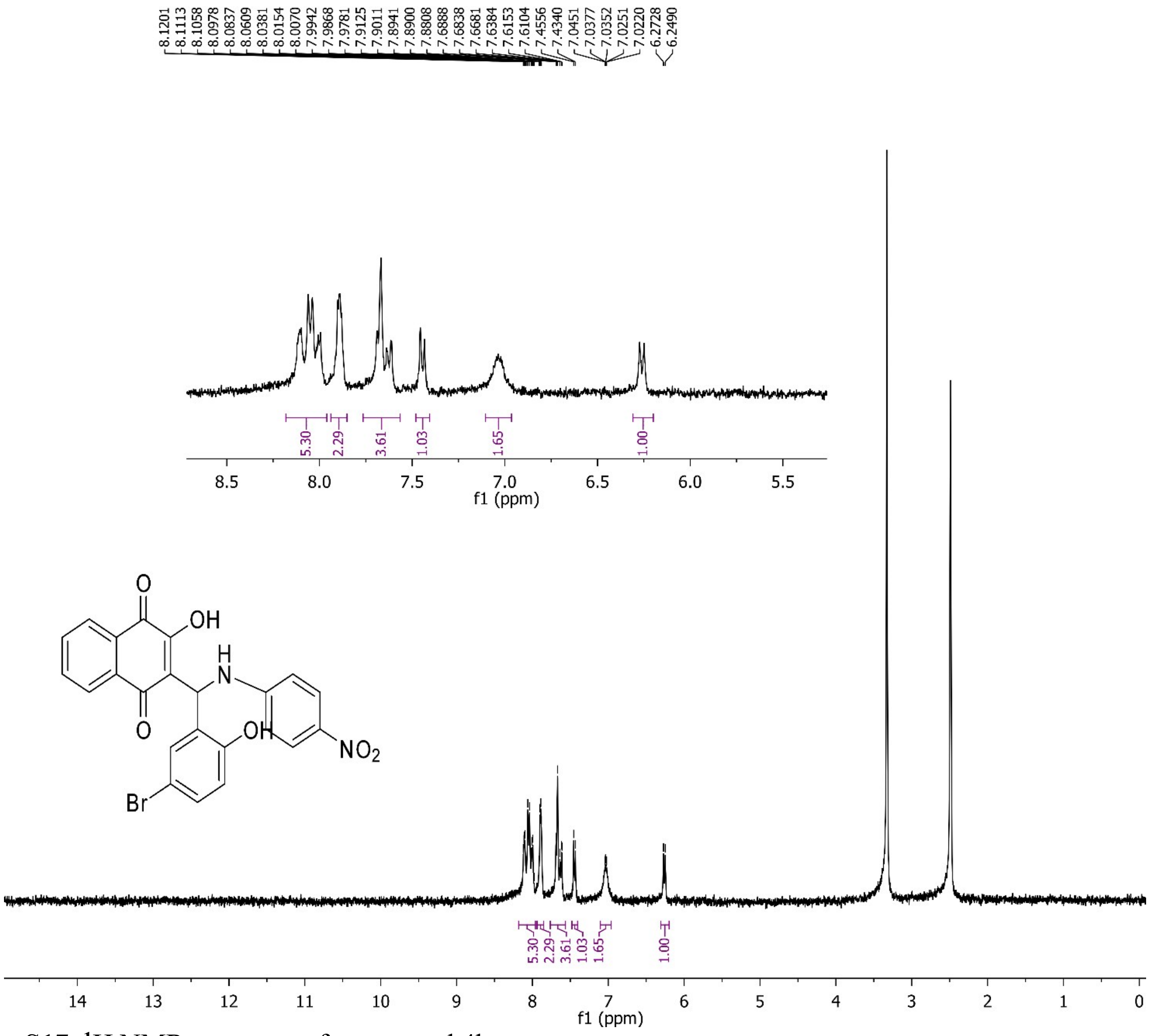

Figure S17. ^{1}H NMR spectrum of compound 4h.

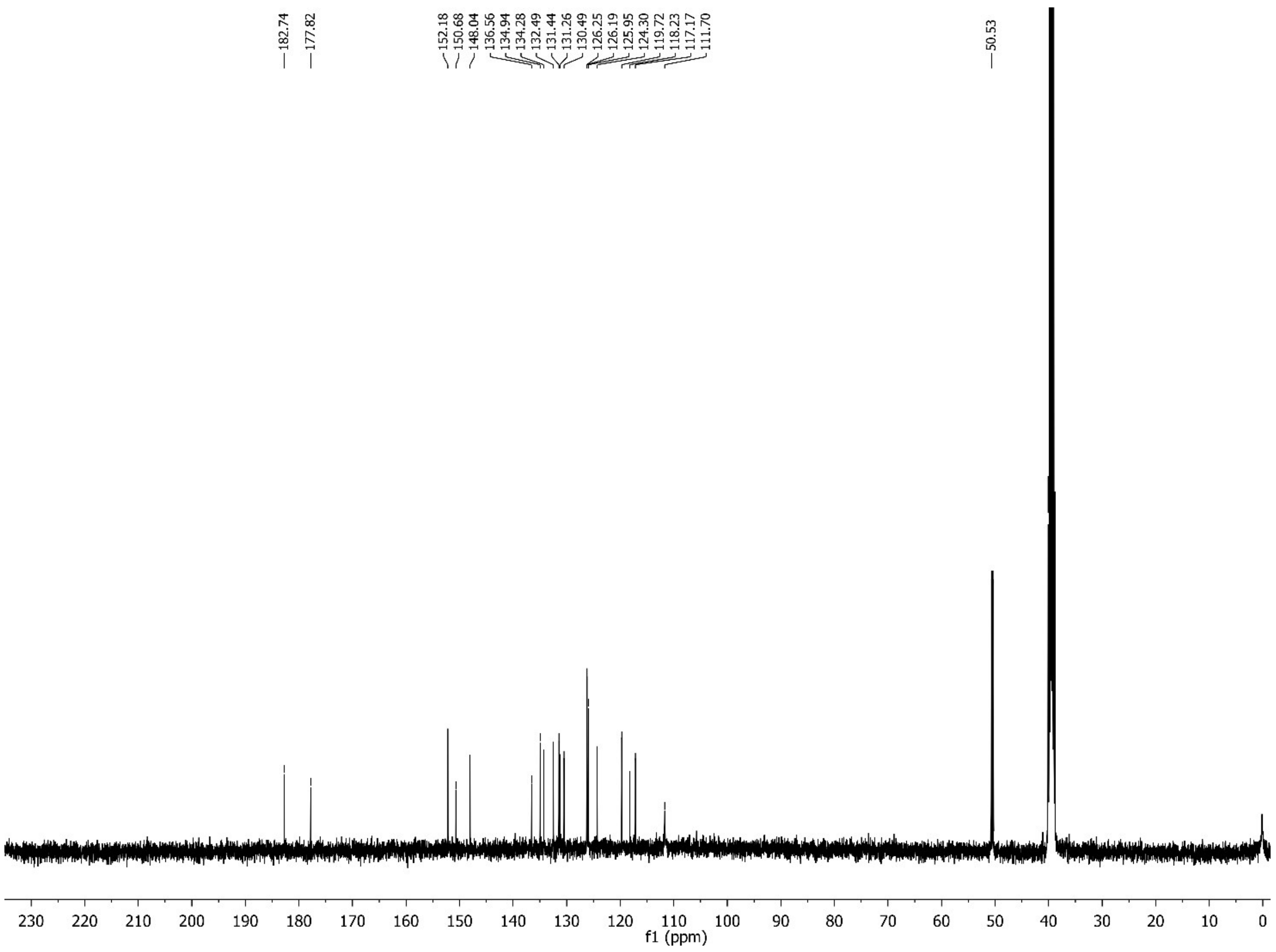

Figure S18. ^{13}C NMR spectrum of compound 4h.

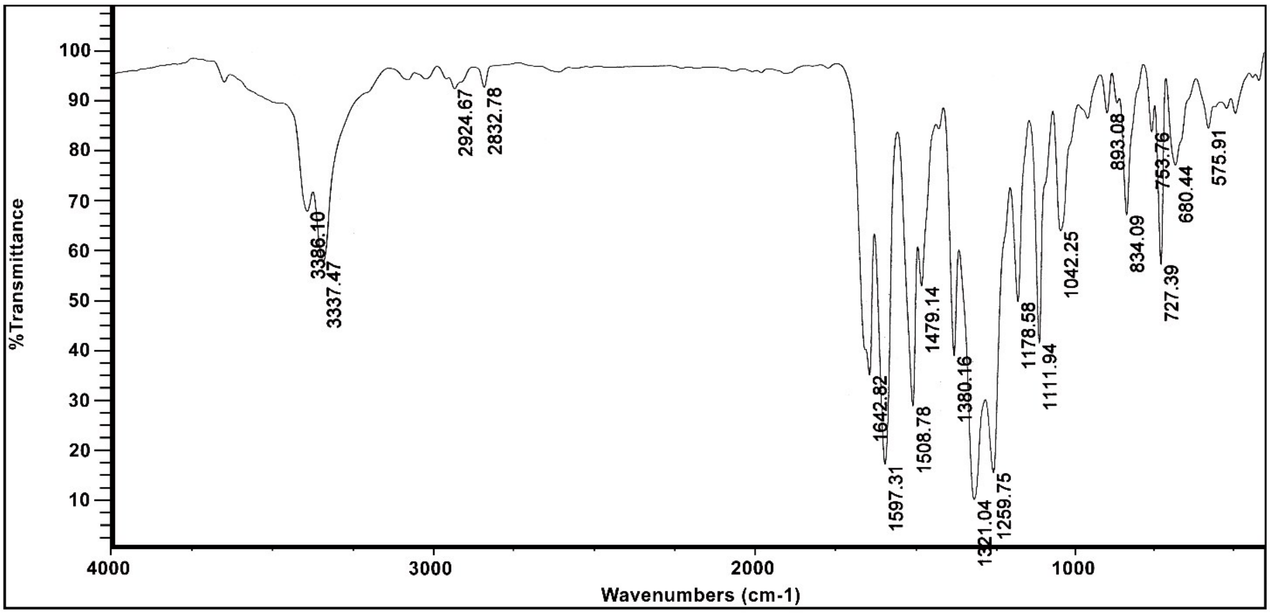

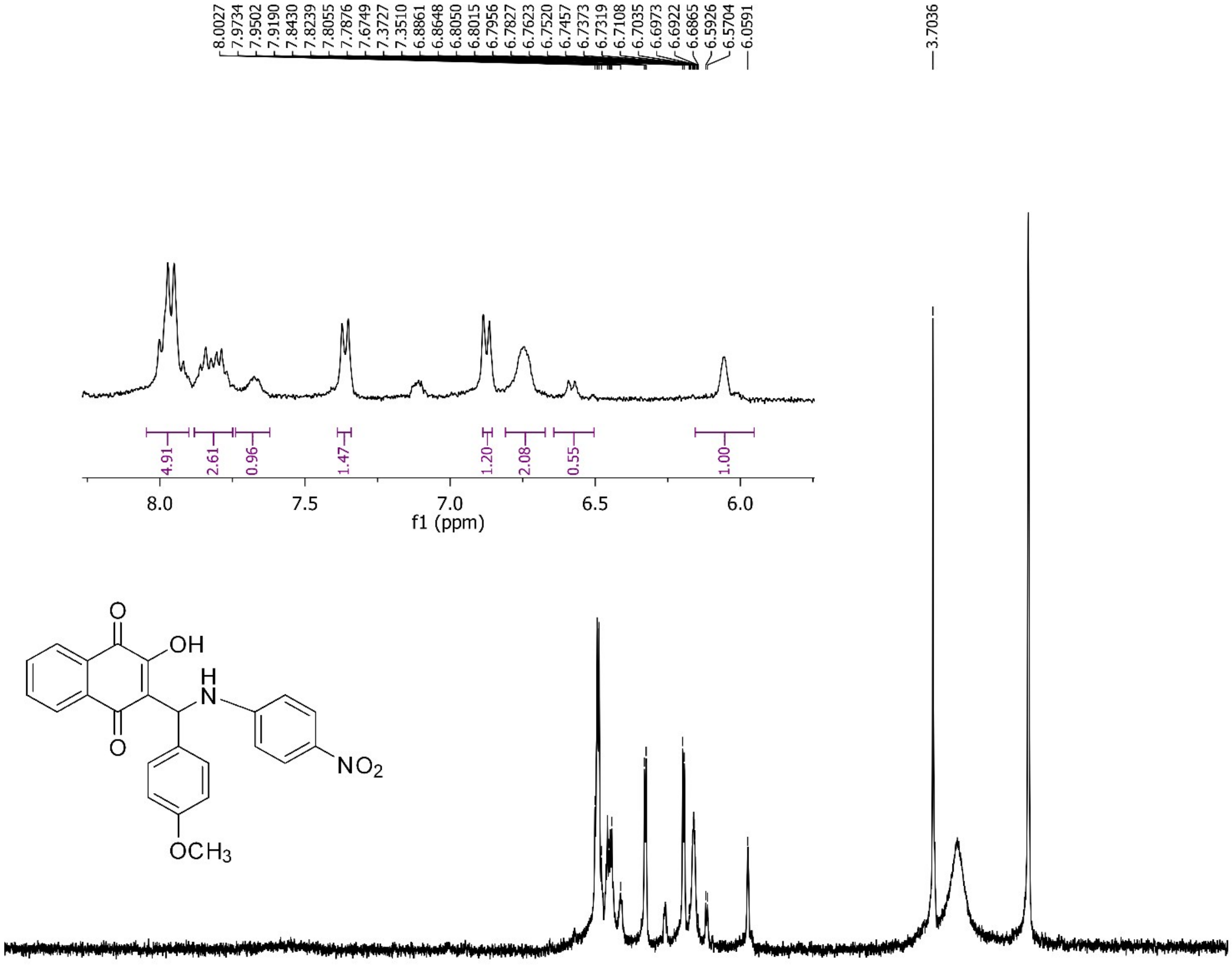

Figure S19. FT-IR spectrum of compound 4i

Figure S20. ^{1}H NMR spectrum of compound 4i.

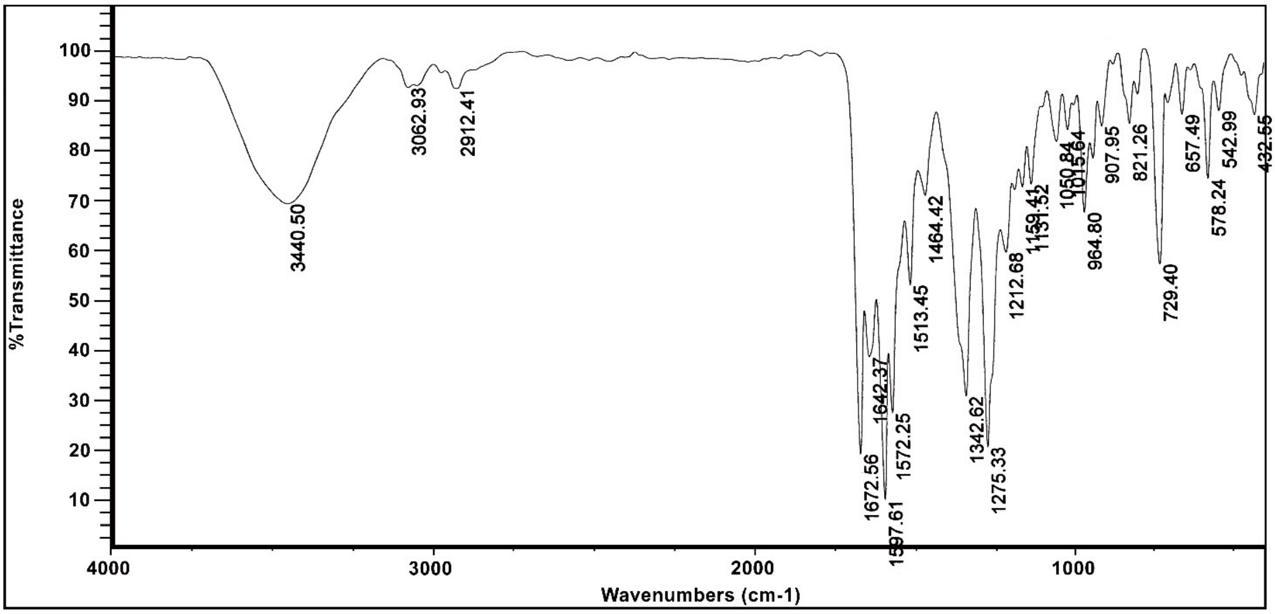

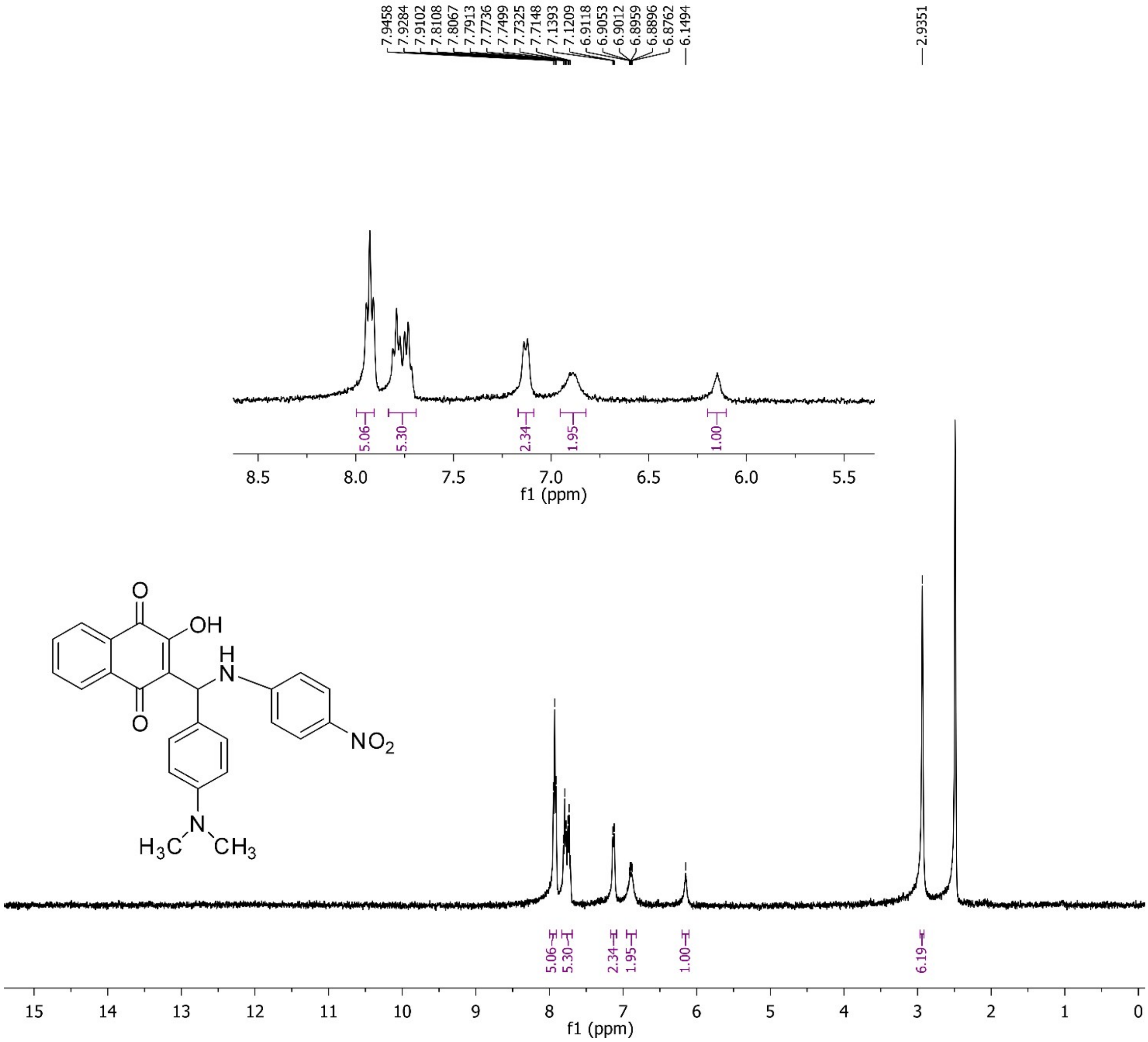

Figure S21. FT-IR spectrum of compound 4j.

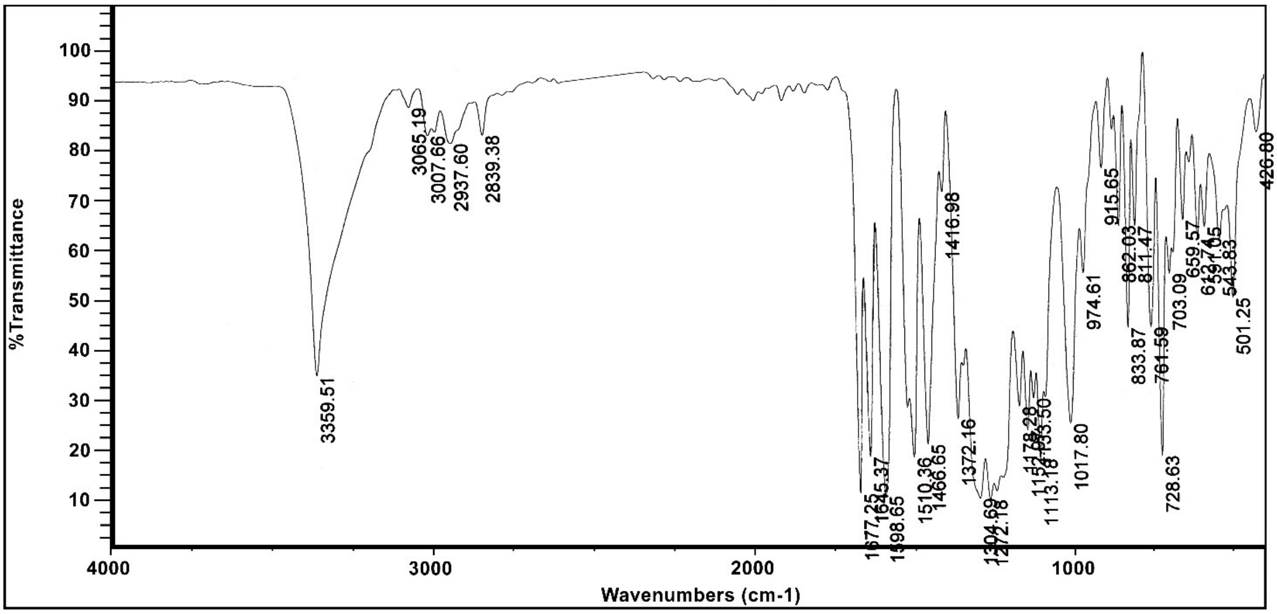

Figure S22. ^{1}H NMR spectrum of compound 4j.

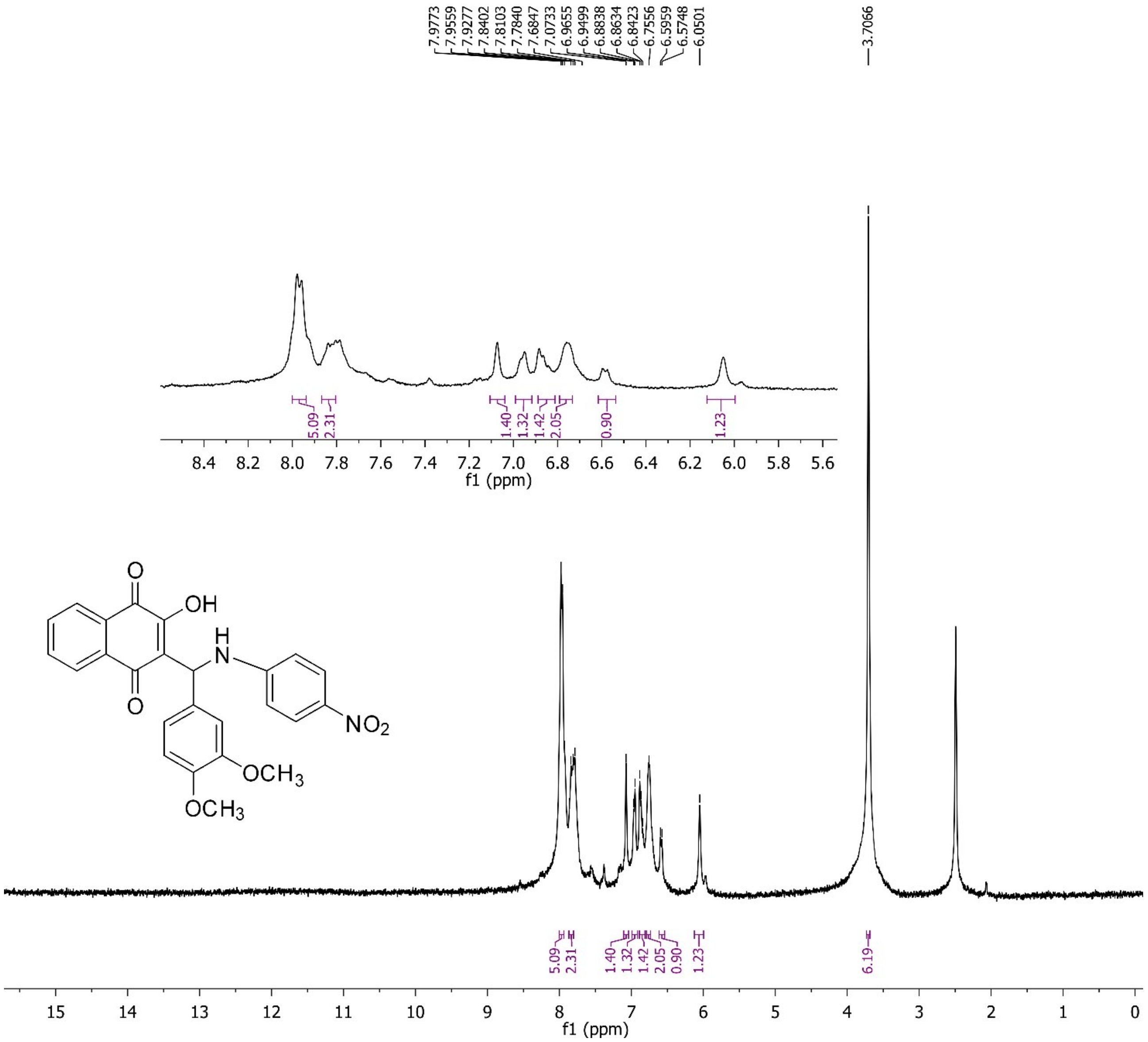

Figure S23. FT-IR spectrum of compound 4k.

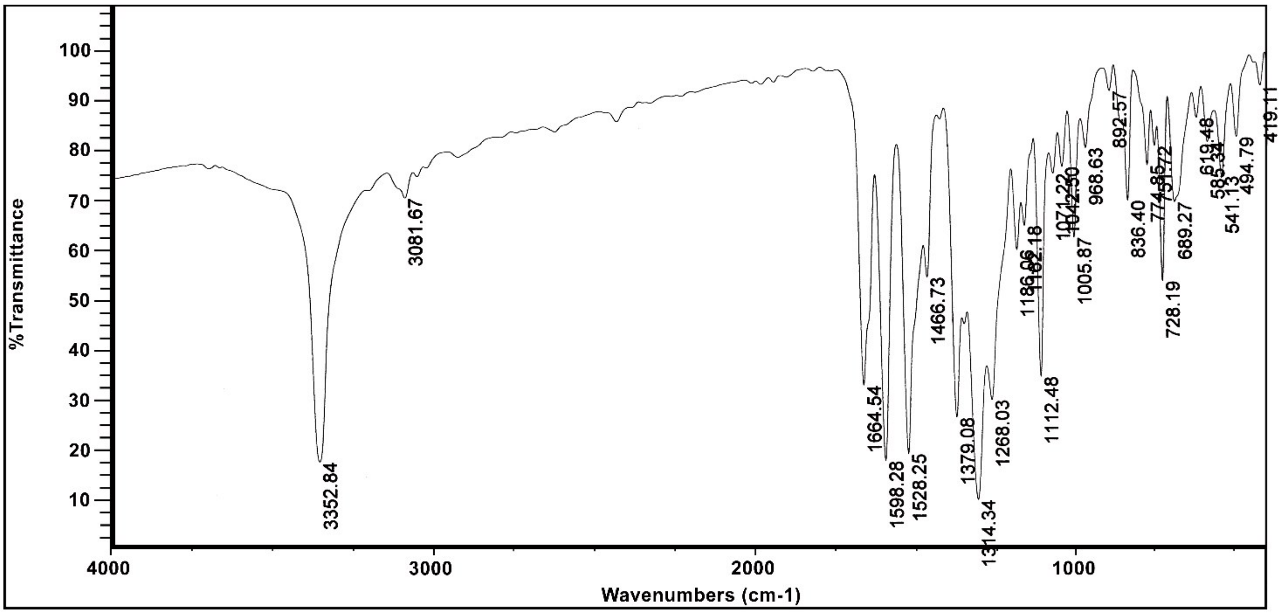

Figure S24. ^{1}H NMR spectrum of compound 4k.

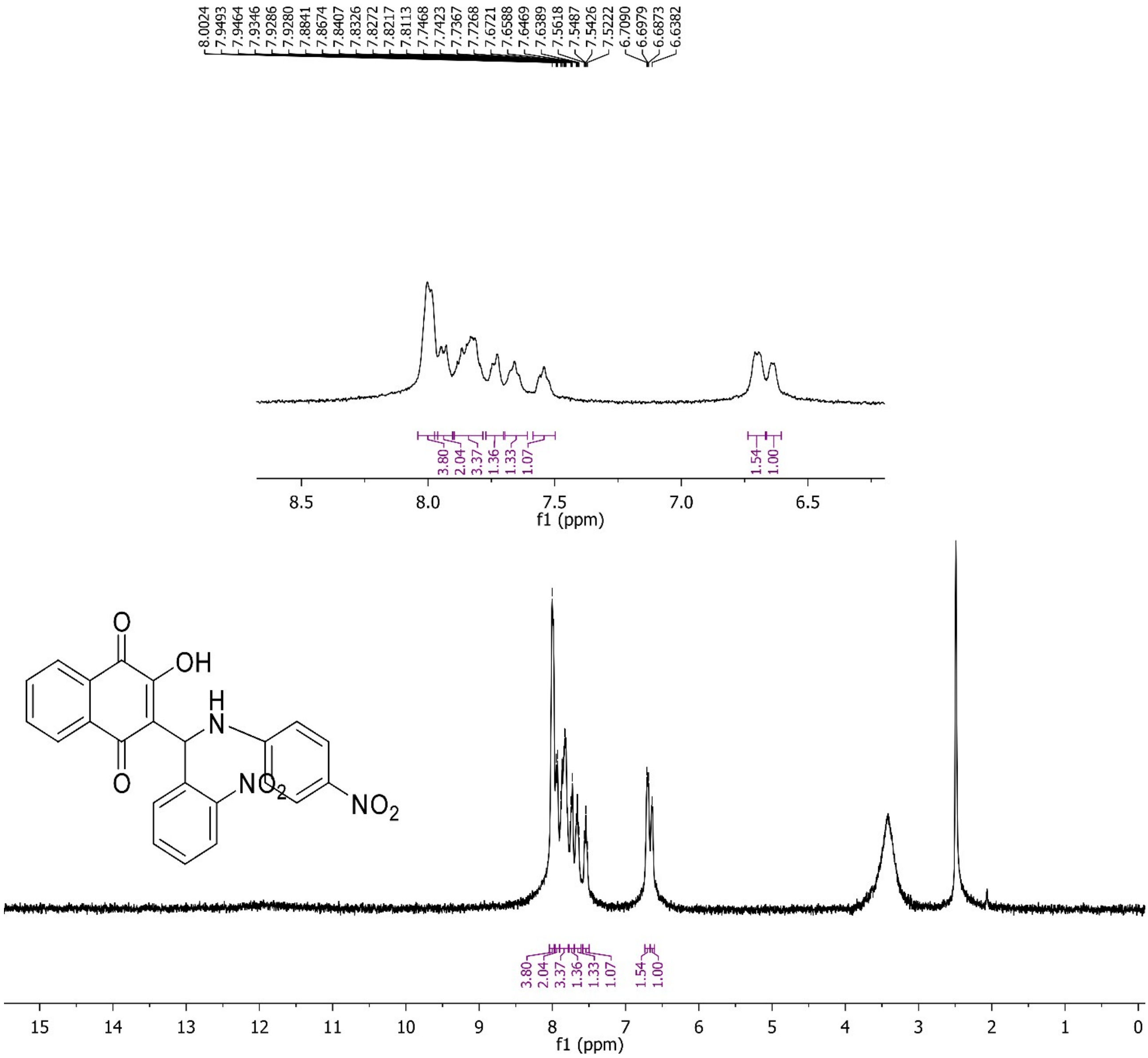

Figure S25. FT-IR spectrum of compound 4l.

Figure S26. ^1H NMR spectrum of compound 4l.
